# Increased Spatial Variability and Intensification of Extreme Monsoon Rainfall due to Urbanization

**DOI:** 10.1038/s41598-018-22322-9

**Published:** 2018-03-02

**Authors:** Supantha Paul, Subimal Ghosh, Micky Mathew, Anjana Devanand, Subhankar Karmakar, Dev Niyogi

**Affiliations:** 1 Interdisciplinary Program in Climate Studies, Indian Institute of Technology Bombay, Mumbai, 400076 India; 2 Department of Civil Engineering, Indian Institute of Technology Bombay, Mumbai, 400076 India; 3 Centre for Environmental Science and Engineering, Indian Institute of Technology Bombay, Mumbai, 400076 India; 4 Department of Earth, Atmospheric, and Planetary Sciences and Department of Agronomy- Crops, Soils, Water Sciences, Purdue University, West Lafayette, IN 47906 USA

## Abstract

While satellite data provides a strong robust signature of urban feedback on extreme precipitation; urbanization signal is often not so prominent with station level data. To investigate this, we select the case study of Mumbai, India and perform a high resolution (1 km) numerical study with Weather Research and Forecasting (WRF) model for eight extreme rainfall days during 2014–2015. The WRF model is coupled with two different urban schemes, the Single Layer Urban Canopy Model (WRF-SUCM), Multi-Layer Urban Canopy Model (WRF-MUCM). The differences between the WRF-MUCM and WRF-SUCM indicate the importance of the structure and characteristics of urban canopy on modifications in precipitation. The WRF-MUCM simulations resemble the observed distributed rainfall. WRF-MUCM also produces intensified rainfall as compared to the WRF-SUCM and WRF-NoUCM (without UCM). The intensification in rainfall is however prominent at few pockets of urban regions, that is seen in increased spatial variability. We find that the correlation of precipitation across stations within the city falls below statistical significance at a distance greater than 10 km. Urban signature on extreme precipitation will be reflected on station rainfall only when the stations are located inside the urban pockets having intensified precipitation, which needs to be considered in future analysis.

## Introduction

Urbanization is a dramatic form of land transformation^1^ which involves the replacement of natural land cover with impervious surface, buildings, and related infrastructures. This form of vertical and spatial land transformation is exceptionally dynamic in India where the urban population is projected to be increased by 404 million from 2014 to 2050^2^. Such extensive urbanization can affect the local to large scale atmospheric composition, surface energetics, water and carbon cycle processes^3^. Especially in the warming climate, this form of urbanization can be considered as an added stress, as its impact becomes further exacerbated^4^. In addition to the local changes, urbanization can also alter the large-scale forcing to bring about changes in the precipitation pattern temporally and spatially. Studies also reveal that, urban land cover can impact far beyond its urban physical boundary^4,5^ such as storm splitting^6^, modifications of large-scale precipitation patterns^7^, modification of storm morphology^8^, or altering the spatial location of storm cells due to increased frictional convergence^9^.

Urbanization affects precipitation pattern in different ways^8,10–16^. Urban land cover acts as a heat source with higher temperature compared to non-urban regions, creating urban heat islands that intensifies precipitation^17^ through additional instability and greater moisture transport; urban emissions produce aerosols that increase the cloud condensation nuclei^18,19^ urban structures work as impediments to atmospheric motion^15^ and create eddy formation with wake diffusion that result into increased precipitation^20,21^. Landsberg^5^ and then Shepherd^7^ summarized an increase of rainfall in the areas within 25–75 km downwind of a metropolis for several US cities.

There is limited number of studies on understanding the impact of urbanization in India on summer monsoon rainfall, which is traditionally believed to be governed and dominated by large scale circulations. Debates exist in the scientific opinion on the impacts of local scale urbanization on large scale monsoon driven extremes. The whole suite of analysis started with Kishtawal *et al*.^22^, which revealed an observed increasing trend in the frequency of heavy rainfall events over the urban and urbanizing regions of India. The analysis was performed with both satellite and gridded rainfall data from Tropical Rainfall Measurement Mission (TRMM) combined rain rate product (3G68)^23^ and India Meteorological Department (IMD)^24^. A series of studies have extended this finding systematically identifying aspects such as increasing spatial variability^25^, increased nonstationarity^26^ and a summary is presented in Niyogi *et al*.^27^ An apparently anomalous conclusion was presented by Ali *et al*.^28^ using the station data from IMD counter arguing that the urbanization impacts are notable only over 4 cities out of 57 Indian cities they considered. Building on this conclusion they projected that the rainfall in future climate with CORDEX regional simulations that do not explicitly consider the feedback from growing urbanization. Similar to this study for India, Mishra *et al*.^29^ have performed a global study and have obtained similar conclusion for precipitation with station data. On the contrary, Ghosh *et al*.^25,30^ showed an increasing trend of spatial variability of rainfall extremes in India that highlight the possible impacts of local processes, such as urbanization. Shastri *et al*.^31^ analyzed the urban impacts on extreme rainfall and have found significant association over the Central Western, Peninsular and North East India. However, that study used gridded rainfall data, which does not explicitly separate out urban and non-urban patterns. A recent study by Singh *et al*.^26^ showed very distinct non-stationarity in the extreme rainfall over urbanizing region compared to the non-urban regions in India. However, this analysis also used gridded rainfall data, mainly due to the unavailability of station data. A research gap thus exists in understanding of the absence of urban impacts in station data of precipitation in India as opposed to that observed in the satellite or gridded data. Note that in studies over the other parts of the world, the stations data has also revealed urban feedback^4,32^.

**Figure 1:**
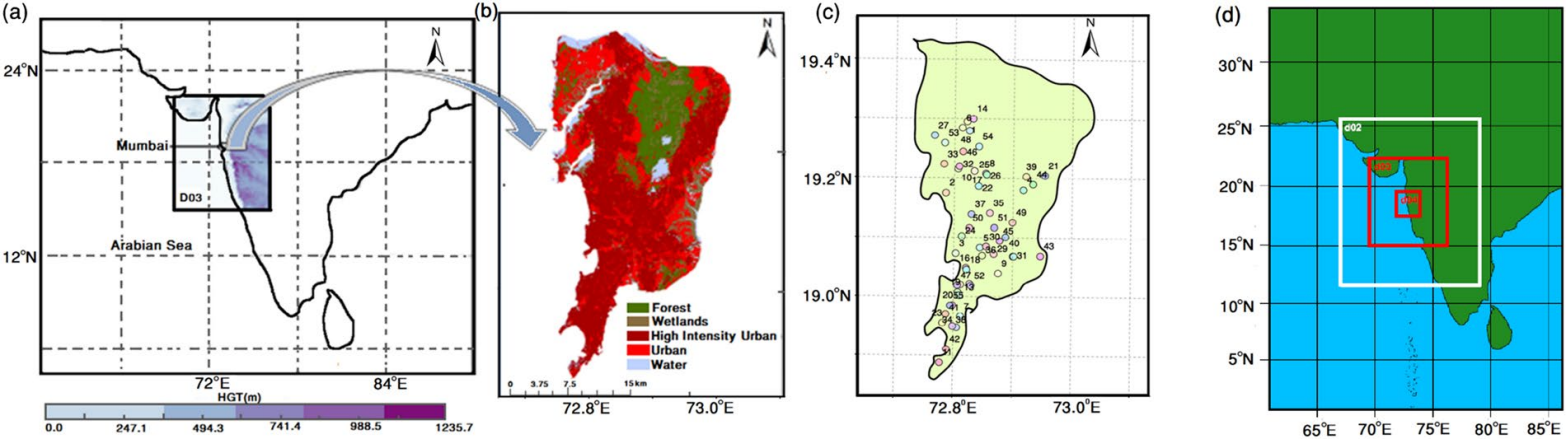
Mumbai City and the domain used for simulations with Weather Research and Forecasting (WRF) model. Location of Mumbai and its land use land cover(LULC) are shows in Fig. (**a**,**b**) respectively. Figure (**b**) represents the LULC of the land area of Mumbai. There are 55 rain gauge stations distributed over the city (**c**). Three nested domains are used for the WRF simulations (**d**). Figure (**a**–**c**) are prepared with ArcGIS 10.1(http://www.esri.com/news/arcnews/spring12articles/introducing-arcgis-101.html). Figure (**d**) is prepared with NCL-6.3 (https://www.earthsystemgrid.org/dataset/ncl.630.html). The shape files of maps are derived from Mumbai Metropolitan Region Development Authority (MMRDA).

We address the above mentioned scientific issues here, with numerical atmospheric simulation of extreme events over Mumbai (Fig. 1(a)), a flood prone and highly vulnerable^33^ coastal city in the western coast of India, at a high resolution (~1 km). The city has a typical land cover of an ‘urban’ region as seen from (Fig. 1(b)). Mumbai is located on the west coast of India (19°07′N, 72°51′E) and receives an average annual rainfall of 2167 mm mainly as summer monsoon rainfall. The megacity has a population of over 21 million^2^ over an area of about 600 km^2^, making it one of the most densely populated cities in the world. The availability of rainfall data from a rain gauge network has facilitated in better understanding of the dynamics of extreme precipitation along with spatial variability. Observations from an extensive dense rain gauge network of 55 Automatic Weather Stations (AWS)^26,34^. Figure 1(c)) were available for 2014 and 2015 summer monsoon rainfall (June, July, August, and September, i.e. JJAS). Though the AWS data have been used in prior studies^26,34^, we have validated the data based on the ‘official’ observed rain gauge station data from IMD. We obtained the observed data for the IMD rain gauge station at Santacruz and computed the correlations with the spatially average rainfall of 5 closest AWS data (within 7 km) at hourly scale (Supplementary Table 1). We obtain high correlation of 0.81 and 0.72 for the extreme events of the year 2014 and 2015 respectively, which presents the partial validation of the data. Based on prior work and model performance tests, ERA-Interim reanalysis data^35^ is used as boundary conditions in the simulations (details in Methods).

We hypothesize that the signature of urbanization in Indian cities may not be uniform all around the city and may be more prominent at few pockets, generated with urban canopy and structure. The stations being analyzed for assessing the impact of urbanization need to be located in those regions to reflect the impact of urbanization on extremes. We test the hypothesis with a fine resolution atmospheric simulation with Weather Research and Forecasting (WRF)^36^ model first coupled with single (WRF-SUCM)^37^, and then multi-layer urban canopy models (WRF-MUCM)^38^. The WRF-MUCM involves the Building Effect Parameterization (BEP), as proposed by Martilli *et al*.^38^. The results are compared with WRF simulations not coupled with UCM (WRF-NoUCM). That is, the base simulation is without explicit urban consideration and the results are reviewed in context of explicit urbanization consideration. The details of the model simulations and configurations are provided in the Methods section. Additionally, while conducting these experiments, we also test the need for the use of convective parameterization (CP) schemes at a fine resolution of 1 km for coastal city like Mumbai (Fig. 1). Our study suggest that there is a need for using a CP scheme even at high resolution to simulate the convective pockets of heavy rains for the Indian region. Convective systems are investigated in detail by Prasad *et al*.^39^. It is also important to assess the model performance in resolving cumulative processes in a terrain characterized by urban canopy and orography. We find that the model performance is poor for the simulations with WRF-NoUCM (Supplementary Fig. 1). The results of the same are presented in the following sections.

## Results

**Figure 2:**
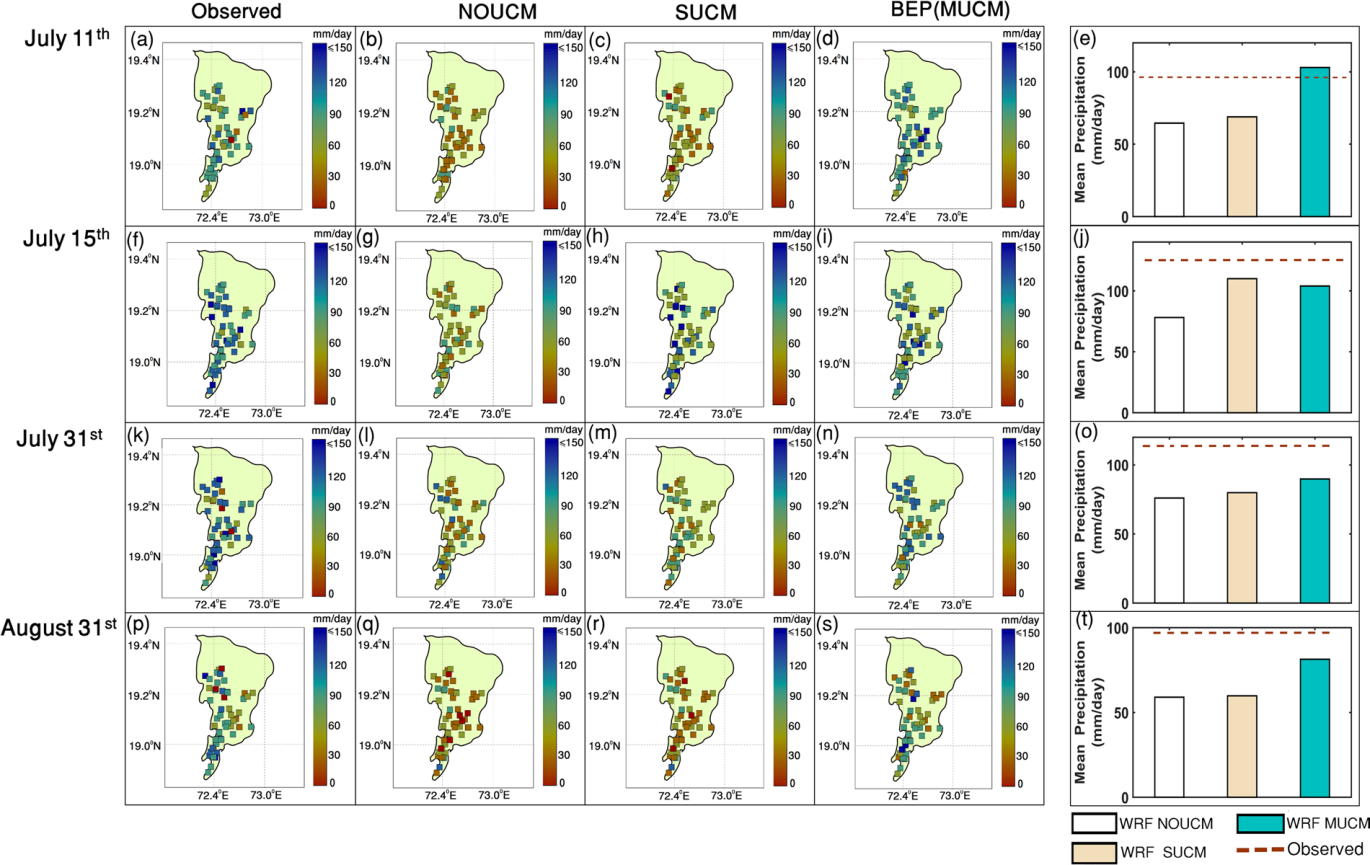
Simulated rainfall for extreme days with WRF. Observed precipitation over 55-AWS stations. Figure (**a,f,k,p**) for July 11^th^, July 15^th^, July 31^st^ and August 31^st^, 2014 and simulated precipitation over the same stations with WRF-NoUCM. Figure (**b,g,l,q**); WRF-SUCM, Fig. (**c,h,m,r**); and WRF-MUCM. Figure (**d,I,n,s**) are presented in first four columns. Spatially averaged precipitation are presented in Fig. (**e,j,o,t**). Figure (**a**–**d**), Fig. (**f**–**i**), Fig. (**k**–**n**) and Fig. (**p**–**s**) are prepared with ArcGIS10.1(http://www.esri.com/news/arcnews/spring12articles/introducing-arcgis-101.html). Figure (**e,j,o,t**) are prepared with Matlab R2016b (https://in.mathworks.com/products/new_products/latest_features.html?s_tid=hp_release_2015b). The shape files of maps are derived from Mumbai Metropolitan Region Development Authority (MMRDA).

We select four different days corresponding to highest rainfall amounts of the year 2014. These events occurred on 11, 15, and 31 July and 31 August 2014. We use observed data from 55 automatic weather stations (AWS) installed by India Meteorological Department (IMD) all over the city for model performance evaluation. The daily rainfall totals for these dates, corresponded to 96.3, 126.9, 113.6 and 96.8 mm, respectively (Supplementary Table 2) and compare the simulated rainfall with WRF-NoUCM, WRF-SUCM and WRF-MUCM (Fig. 2). In the figure, left-most panel shows the observed data at the weather stations while the successive panels show simulations from WRF-NOUCM, WRF-SUCM and WRF-MUCM respectively over the grids that contain the stations. We find that the rainfall simulated with explicit consideration of urban feedbacks is consistently higher as compared to the WRF-NoUCM run. Further, WRF-MUCM rain amounts are generally higher for all the days as compared to the same by WRF-NoUCM and WRF-SUCM. During July 15, 2014, the rainfall simulated by WRF-SUCM is slightly higher than the WRF-MUCM; however, both of them are higher than that simulated by WRF-NoUCM. The differences between WRF-SUCM and WRF-NoUCM simulations support the notion of the intensification in precipitation due to urbanization. WRF-MUCM further considers more specific impacts due to the pattern of urban structures, canopy, buildings, and road network orientation and these subgrid scale features indeed create atmospheric perturbations and disturbances in the boundary layer in response to these patterns. WRF-NoUCM simulates low rainfall consistently all over the city. Indeed all the simulations underestimate heavy rainfall except for the extreme events on July 11^th^, 2014, when WRF-MUCM has almost similar amount of rainfall as observed over the city (spatially averaged). These differences are expected as urban morphology data are not available and other physics such as building energy as well as urban aerosol interactions is not considered in these simulations (primarily because of lack of high resolution emission inventory data). The rainfall observations shown in the left panel of Fig. 2 shows huge spatial variations (except July 15, 2014) and interestingly some of these are relatively well captured by WRF-MUCM. Note that, because of the rare occurrences of extremes and low availability of spatially distributed rainfall data from AWS over the city, the sample size remains small. To reconfirm the intensification of extremes due to urban canopy structure, we perform the simulations for 4 more extreme days that occurred during 2015 (Supplementary Fig. 2). Daily observed rainfall for all the four extreme days during 2015, viz., June 18, June 20, June 21 and June 23rd, are 117 mm, 78.69 mm, 80.2 mm and 101 mm respectively. Our key conclusion of intensification of extreme rainfall due to urban canopy remains unchanged, and in fact is further strengthened, with the sets of simulations from 2015. We also find lower error in the simulated precipitation by WRF-MUCM as compared to the same by WRF-NoUCM and WRF-SUCM. We find intensification of extreme rainfall due to urban canopy over the majority of the locations of Mumbai (Supplementary Fig. 3) even at hourly scale for both the years 2014 and 2015, and such increases are statistically significant at the level of 0.05. We further test the need for performing simulations at a very high resolution of 1 km for urban region. We have computed the absolute errors between simulated and observed rainfall data for the extreme events that happened during 2014 and 2015 over Mumbai. The errors are compared between the simulations with spatial resolutions of 27 km, 9 km, 3 km and 1 km (Supplementary Fig. 4). We find least error with the simulations performed at a spatial resolution of 1 km and this justifies the need for simulations at a very fine resolution for urban studies. The rainfall amounts at different stations indicate the existence of high spatial variability and this is likely the basis for creating the within city urban rainfall modifications and is tested further.

**Figure 3:**
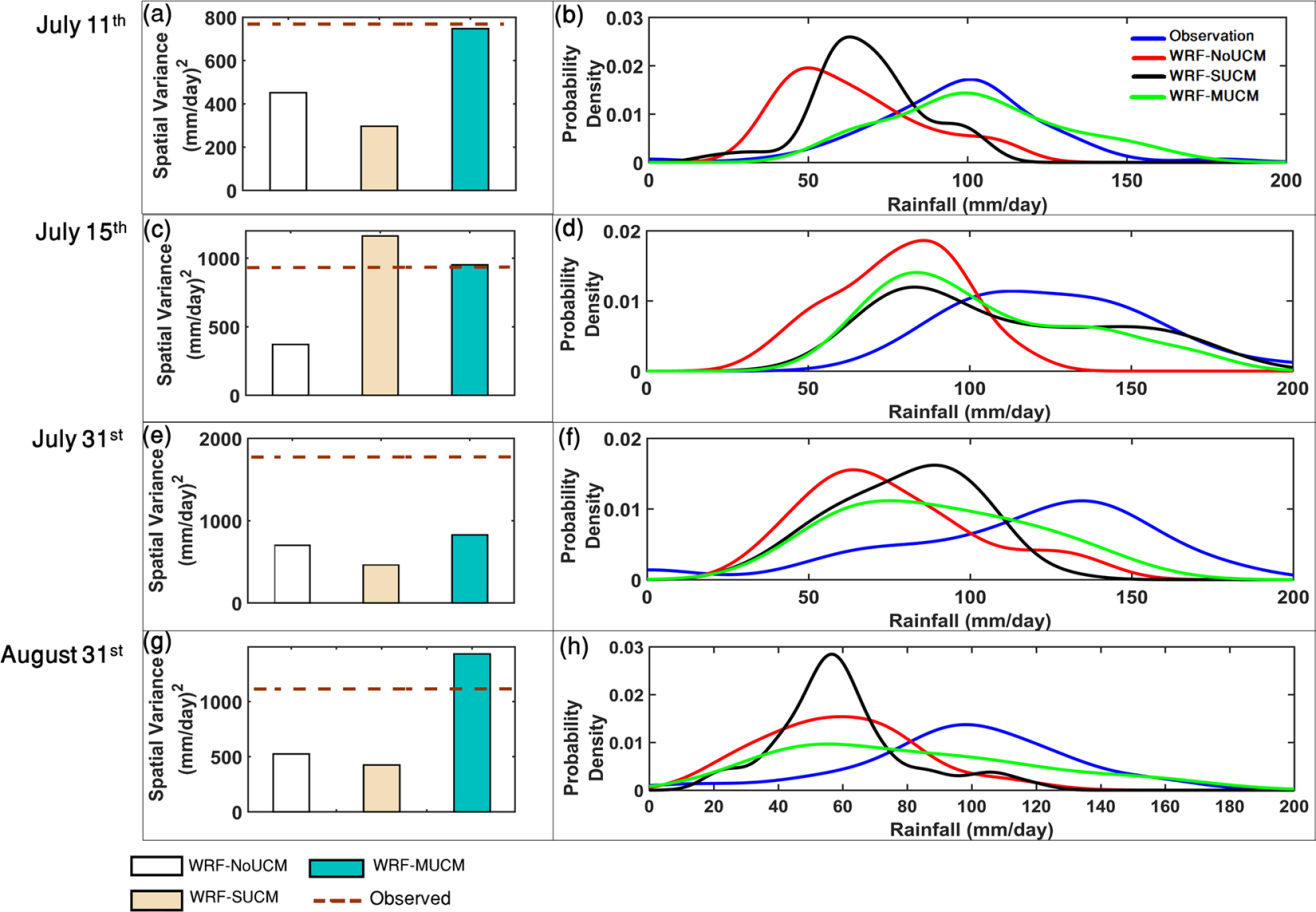
Spatial variance of observed and simulated rainfall across stations. Figure (**a,c,e**,**g**) and their representative probability distribution functions. Figure (**b,d,f,h**) for all the four extreme days. All the figures are prepared with Matlab R2015b (https://in.mathworks.com/products/new_products/latest_features.html?s_tid=hp_release_2015b).

The spatial variability of rainfall amount across stations is presented with the spatial variance (Fig. 3, left panel) and spatial probability density functions (pdfs, Fig. 3, right panel). The WRF-NoUCM simulates low spatial variations in rainfall as compared to the simulations with urban feedback. Comparing the results from this numerical experiment clearly shows that urban feedback produces an increase in the spatial variation of rainfall across different parts of the city, intensifying the rainfall non-uniformly within the urban space. Therefore, WRF-MUCM simulates high spatial variability of extreme rainfall in Mumbai compared to the simulations by WRF-NoUCM and WRF-SUCM. Indeed in support of the perspective that the spatial variability translates to rainfall intensification from the city, on July 15^th^, 2014, WRF-SUCM simulates high spatial variability though the spatial variation simulated by WRF-MUCM is closest to that from the observed data (Fig. 3(c)). On the other hand, for July 31^st^ 2014, all the simulations underestimate the spatial variability significantly, apart from WRF-MUCM, which simulates high spatial variability. These numerical experiments lead to the conclusion that urban form and layout cause spatial variability of rainfall and this in turn primes the atmospheric setting for urban rainfall intensification.

**Figure 4:**
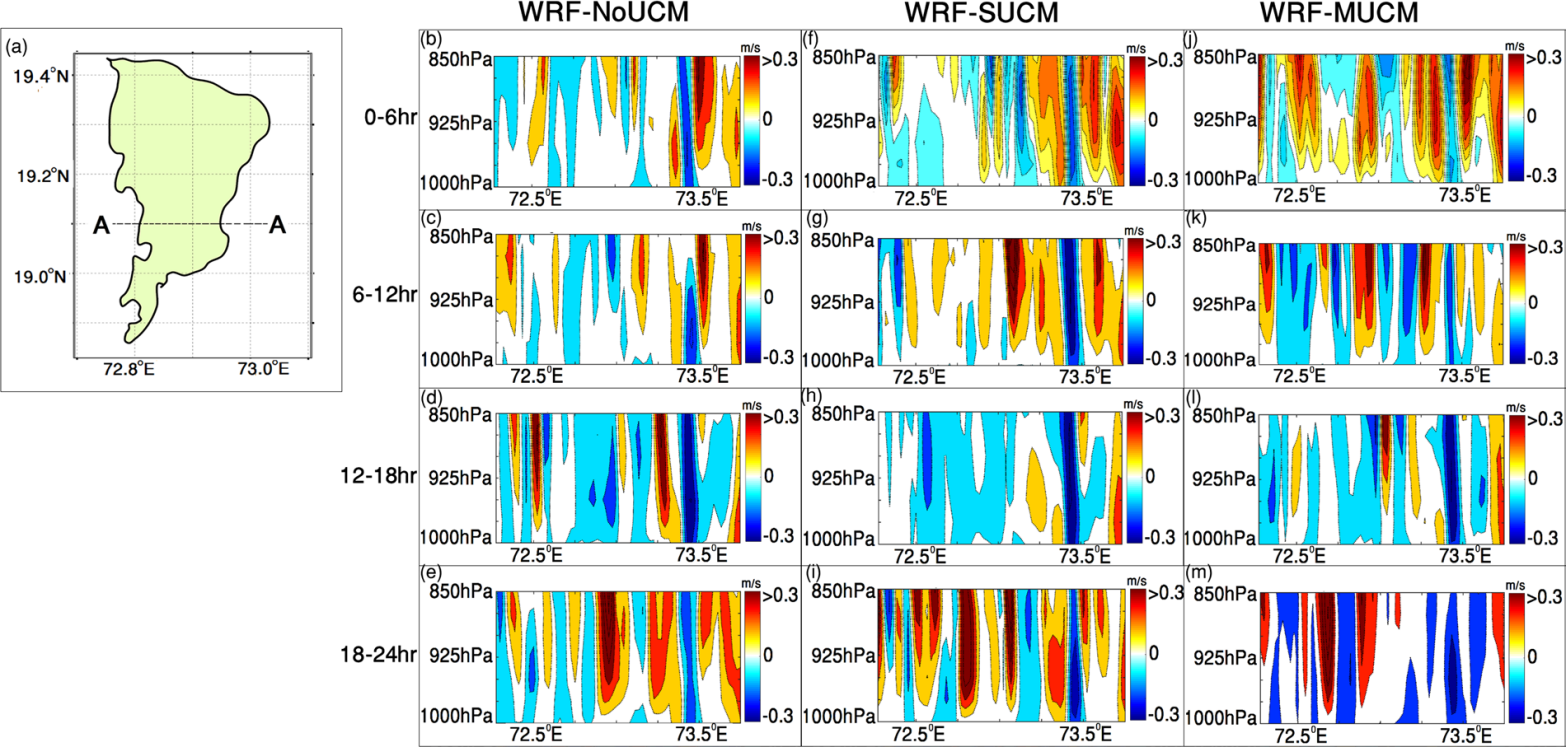
Cross-section considered for the analysis of atmospheric instability is presented in Fig (**a**). Simulated instability at 6-hr interval for WRF-NoUCM Fig. (**b**–**e**), WRF-SUCM (Fig. (**f**–**i**)) and WRF-MUCM. Figure(**j**–**m**) during July 11^th^, 2014 are presented. Figure 4(a) is prepared with ArcGIS-10.1 (http://www.esri.com/news/arcnews/spring12articles/introducing-arcgis-101.html). Figure 4(b–m) is prepared with Matlab R2015b (https://in.mathworks.com/products/new_products/latest_features.html?s_tid=hp_release_2015b). The shape files of maps are derived from Mumbai Metropolitan Region Development Authority (MMRDA).

To further understand the mechanism, we plot the vertical velocity at a cross section (Fig. 4(a)) for different simulations performed for 11^th^ July, 2014. The cross section extends from west to east over the middle of the city with high built-up area. The results presented in Fig. 4 show that the instability (as defined by vertical velocity) is lower when explicit urban effects are considered in the simulations. The WRF-SUCM simulation shows that the unstable conditions prevail in the boundary layer and they are intensified at the eastern side resulting an eastward pattern (Fig. 4(f)) during 00–06hrs (local time). The WRF-MUCM shows localized pockets of updrafts and downdrafts contributing to the increased instability due to the buoyant plumes. These patterns evolve and show a heterogeneous pattern (Fig. 4(j)) at a shorter distance resulting in increased spatial variability. The same is also obtained during 06–12 hours (local time). During 18–24 hours, the WRF-MUCM in particular shows high vertical velocity and strong spatial variability with updrafts and downdrafts contributing to the atmospheric flow pattern over a short distance, which is not there in any other simulation. Extremely high variation of vertical velocity within a small distance is the result of heterogeneity due to urban structures, such as building canyons, impervious surfaces, roads and the resulting impacts on surface energy balance and overlaying atmosphere are captured within the WRF-MUCM simulations.

**Figure 5:**
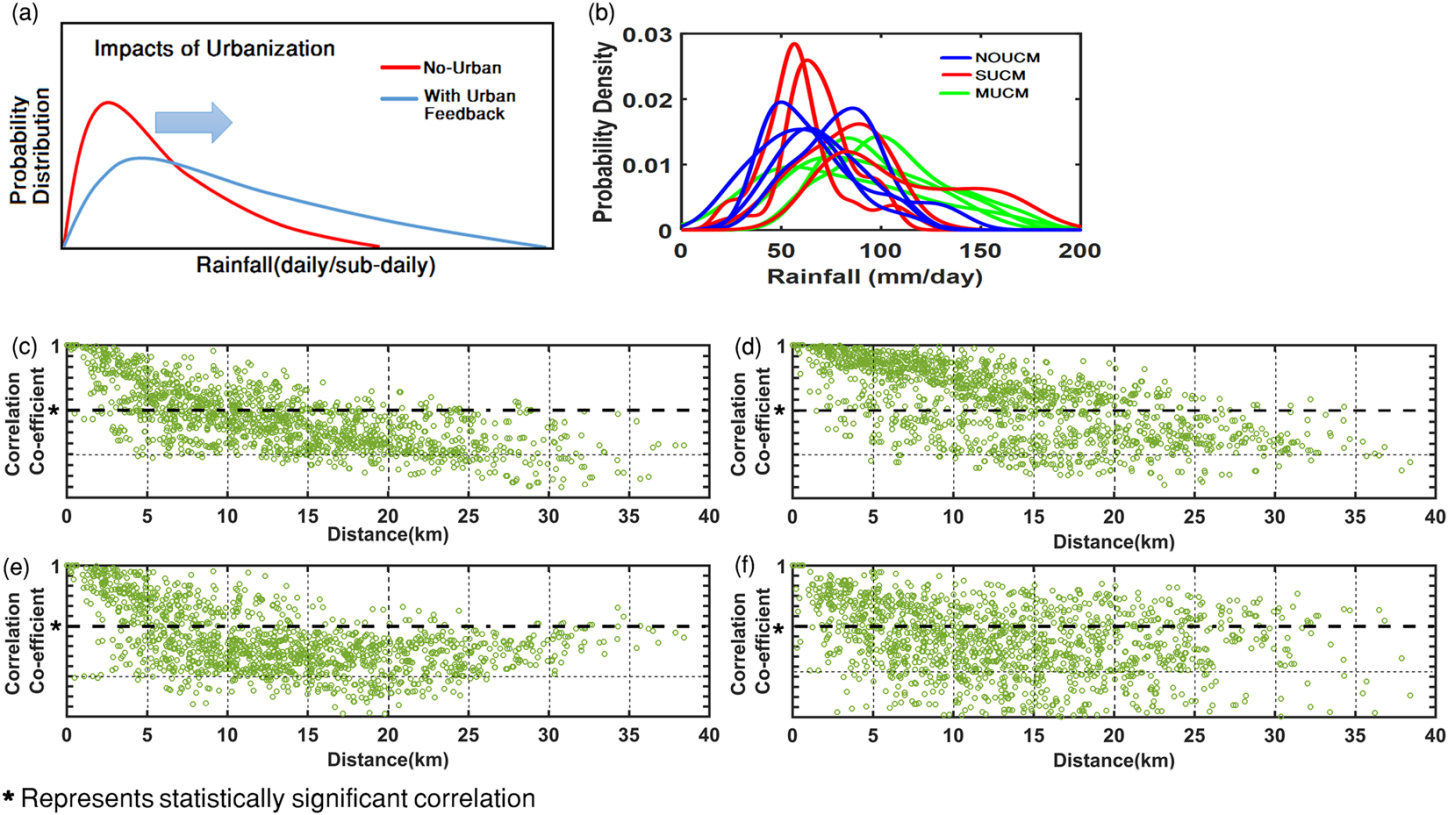
Schematic of urban impacts on the spatial variability of precipitation over a city is presented in Fig (**a**). Model simulations for Mumbai are presented in Fig. 5(b). The PDFs represent the spatial variability. Figure 5(c–f) represents scatter plot between cross correlation of precipitation and distance between the AWS stations for July 11, July 15, July 31 and August 31 respectively. Figures are prepared with Matlab R2015b (https://in.mathworks.com/products/new_products/latest_features.html?s_tid=hp_release_2015b).

The results suggest that, while conventionally urbanization in terms of land cover extent (fraction) is considered to be an influencing factor for the intensification of rainfall; here we find that such intensification may not be apparent everywhere in the city as it is due to localized effects with intensification of rainfall at several urban pockets. The impacts of urbanization will be distinctly visible in terms of increase in spatial variability. We conclude that urbanization will lead to flattening of the probability density function (PDF) representing the spatial variability of precipitation at the stations distributed over a city with a heavy tail (Fig. 5(a)). The PDFs are obtained using the simulated rainfall corresponding to the AWS station locations. The simulations show similar trend and a progressive change from peaked PDFs to flattened heavy tailed PDFs are noted with simulations from WRF-NoUCM to WRF-SUCM and WRF-MUCM (Fig. 5(b)). The PDFs are obtained for four (4) representative days selected as extreme days. We find an increase in the sparseness of intensity of rainfall values across stations and that results in an increase in spatial variability. Spatial variability is ideally a measure of sparseness of data from different stations in the region (here, city of Mumbai). With the increase in sparseness, the width of the base of PDF increases and the peak of PDF decreases. In Fig. 5(b) we find that WRF-MUCM results into the PDFs with high base and low peak (flattening of PDF). Consideration of BEP in WRF–MUCM results into intensification of extreme rainfall at some of the urban pockets, and non-uniformly over the whole city. This attributes to formation of spatially varying instability. We also plot the changes in wind at 850 hPa (m s^−1^), moisture convergence(s^−1^) and surface fluxes (W m^−2^) due to incorporation of MUCM in WRF simulations (Supplementary Fig. 5). Results indicate that the impacts of urbanization on these meteorological variables are either statistically insignificant or very low.

The above results further confirm that the changes in the simulated rainfall and the increase in spatial variability of extreme precipitation over urban region is due to the atmospheric instability arising from urban morphology such as structures of road, slum area, mid-rise buildings and high rise buildings and not just the urban cover. The increase in spatial variability due to urbanization highlights the need for an urban specific guidelines for setting up meteorological stations, which is at present not in practice particularly for Indian cities. Urban signature on extremes should be captured based on distributed rainfall pattern over the city rather than the traditional method of considering single stations set up in the city. We further obtain the cross correlation of simulated (WRF-MUCM) precipitation among stations and plot the same with distance between the stations (Fig. 5(c–f)). We find that when the distances between the stations go beyond 10 km, majority of cross correlation values (more than 50%) go below statistical significance. This was noted for all the extreme days except July, 15, 2014. This further point that to study urban rainfall patterns, the rainfall station spacing should be within every 10 km. This conclusion needs to be further tested with multiple simulations and this may be considered a potential area of future research.

Regional climate simulations at a finer resolution (less than ~10 km) are presumed to resolve the convective processes and hence the recommended parametrization methods are not required^40–43^ on the other hand, there are studies which suggest, to overcome under-resolved convection at high (<4 km) resolution, separate explicit convection schemes are essential^42,44–49^. The motivation also comes from the recent works of Prasad *et al*.^39^ over India. We tested the same to assess if the feedbacks are not being captured in some of the model runs because of the convective schemes. Hence, experiments were performed with and without convective parameterization representation within the model. Experiments were conducted with Kain–Fritsch (KF) scheme^50^ building off the results from prior studies, which conclude an overall good performance when using this scheme over the Indian region, especially for simulating convective storms^51^. We also highlight that in an urban environment, to capture turbulence occurring at finer spatial resolution (eddies generated within urban canopy, which subsequently impact convection generated within urban boundary layer), may require building-resolving large-eddy simulations (LES) models (typically run at 100 m grid spacing)^52^ coupled with mesoscale model. At the same time, there are earlier studies^53^, which suggest that discrete, isolated convective cells evolving within sub-grid scale convective processes cannot be explicitly resolved by cloud micro-physics alone. Hence, we use explicit convective parameterization scheme (CP), alongside BEP(WRF-MUCM), which seeks to resolve the convective processes at local urban scale. Our results indicate that even for 1 km grid spacing, considering CP scheme adds value when WRF is coupled with MUCM, in terms of reduction of errors (Supplementary Fig. 1). Except July 31^st^, 2014, all the other three days show lower errors at individual stations when convective scheme is used. All our analysis has been performed with the same convective scheme.

To further analyze how the urban form is contributing to the formation of the mesoscale instabilities and hence the rainfall pockets, we performed experiments with multiple street orientation angles that are possible in WRF-MUCM. We find that errors are minimum for angle 0–0 and angle 0–90 (Supplementary Fig. 6). The analysis is performed for the first day of extremes, i.e., July 11, 2014. We further present the PDF of spatial variability of simulated precipitation for different street orientation (Supplementary Fig. 7). We find irrespective of orientation, the WRF-MUCM produces high spatial variability of precipitation with reference to WRF-NoUCM. This further generalizes that urbanization leads to increase in spatial variability of extreme rainfall due to urban form; however, the intensification of rainfall may occur at different places depending on layout of urban form and morphology such as building canopies, heat sources, and road network.

We performed additional analysis for low intensity rainfall days (Supplementary Fig. 8). Simulations are performed for moderately low (40–50 mm/day), low (20–30 mm/day) and extremely low (<5 mm/day) intensity rainfall events (Supplementary Fig. 8(a–r)). We find that light intensity rainfall events are also simulated reasonably well by the WRF-MUCM model without any wet bias.

We also perform simulations with multiple physics options in WRF for two of the extreme events (July 11^th^ and July 15^th^). We consider combinations of five different cumulus schemes^50^^,S4–S9^ and two planetary boundary layer schemes^54^^,S10^ (Supplementary Table 3) for both WRF-MUCM and WRF-NoUCM schemes. We find intensified extreme rainfall in the simulations when urbanization is considered (Supplementary Fig. 9(a–b)). Our findings are consistent across all the parameterization schemes.

We also compute the spatial correlation for all the three simulations; WRF-NoUCM, WRF-SUCM and WRF-MUCM with respect to the observed data. We select 6-hourly data for this comparison, and find WRF-MUCM outperforms the other two. WRF-SUCM simulations are better than WRF-NoUCM and WRF-MUCM simulations are slightly better than WRF-SUCM (Supplementary Fig. 10(a–c)). These results highlight that urban form and morphology rather than urbanization alone is important for the creation of mesoscale instabilities which translate into highly spatially variable rainfall patterns in and around the city.

## Conclusions

Using a network of observations available around Mumbai and a series of high resolution (1 km) model runs for cases corresponding to urban precipitation at the coastal city of Mumbai with different urban models, several consistent results and conclusions can be drawn.

1. Urbanization leads to significant increase in spatial variability of monsoon rainfall within the city and this is due to the generation or reorganization of instabilities at a local scale (~10 km). The gradients in these instabilities become the locales for intensifying rainfall patterns and lead to extreme precipitation at few urban pockets. This increase in spatial variability of precipitation extreme within the city can be considered as another quantifiable signature of urban impacts on precipitation.
2. Urbanization leads to intensification of extreme precipitation and this is notable in several urban pockets in response to the within city instabilities. Indeed detecting such urban induced intensification in station data would mean that the station has to be located in these pockets. This would be an important reason that some studies using stations data have been unable to identify urban signature with station rainfall. Since the mesoscale instabilities are in response to the dynamic meteorological conditions, the location of these intensity pockets will also change.
3. Because the urban rain hotspots will be variable, either high resolution insitu monitoring or radar datasets continue to be the best effective means of observing and studying the urbanization impacts on rainfall. Individual station data based analysis would very likely lead to an erroneous conclusion of lack of urbanization impacts on rainfall if those stations are not at a sufficient high spatial density.
4. At present, in India, there is no specific guideline for setting up urban meteorological stations. Increase of spatial variability of rainfall in a city shows the need for the same. As for example, we find that the statistically significant correlation mostly dissipates in Mumbai at a scale larger than 10 km due to spatial heterogeneity and this needs to be considered while setting up the stations. The station data may further be blended with satellite data to develop higher resolution datasets.
5. The WRF-MUCM simulates the extreme rainfall better than WRF-NoUCM and WRF-SUCM. This shows the role of the patterns of urban form such as structures, roads rather than urbanization alone in the simulation of instability and resulting intensified extreme rainfall. At present, the operational weather forecast in India does not consider multi-layer urban canopy model and incorporation of the same will aid improving of the forecast skill of urban precipitation extremes.

The limitations of the study are the followings:

1. The position of built-up areas and green fractions is representative of the present condition in Mumbai. Consideration of exact urban building morphology and road structures was not done due to lack of availability of such data. Incorporating these features would likely further improve the model simulations. This may be incorporated with the newly developed World Urban Database and Access Portal Tools (WUDAPT) framework^55^.
2. The number of events considered for the present analysis is limited to four for 2014 (with four more for 2015), due to the constraints of data availability and computing requirements. Use of multiple seasons with high sample size will make the analysis robust. The uncertainties associated with limited experiments and model deficiencies have not been addressed in this work and is a potential area of future research.
3. The hypothesis of intensification of urban extremes needs to be further tested with multiple case studies across different cities to make it more generalized and this can be a potential area of future research.
4. Various gaseous and particulate emissions within the urban environment may affect precipitation; however, WRF-MUCM cannot explicitly consider the same. It may require a separate model like WRF-Chem to be coupled with WRF-MUCM. Lack of high resolution emission inventory data limits our ability to consider the impact of gaseous pollutants in urban region and this may be considered as the potential future area of research.

The present study concludes that the urban rainfall and convection manifests as a feedback of turbulence leading to vertical motion, surface energy flux gradients. The changes in the turbulence and mean fields lead to the modified meteorological conditions leading to intensified rainfall.

## Method

### Model Configuration

This study employs the WRF-ARW^36^ (WRF version-3.6.1) to simulate four typical extreme rainfall events that occurred in the Mumbai city over the year 2014 (and 2015). These numerical experiments were conducted to capture the association between urban rainfall events and the humid, convective, unstable circulation over urbanized regions.

The horizontal resolution of the one-way nested domains D01, D02, D03, D04 are 27 km, 9 km, 3 km and 1 km, respectively as shown in Fig. 1(d). The model uses 42 vertical pressure levels starting from 1000 hPa with the 16 terrain-following sigma levels in the lowest 1 km. The 30 s MODIS land cover data was used for the numerical representation of land categorization in the model. The ERA-Interim reanalysis data^35^ of resolution 0.75° × 0.75° provided by ECMWF (European Centre for Medium-Range Weather Forecasts) has been used for providing initial and lateral boundary conditions to the model. Based on preliminary sensitivity tests the following physics options were found to give the best results for the study area.

The Bougeault and Lacarrere (BouLac) scheme^54^ is used for representing the planetary boundary layer. The Eta surface-layer scheme^56^ is used to represent surface layer physics. The Thompson scheme^57^ is used for representing the microphysics; while the Kain–Fritsch (KF) convective parameterization scheme^50^ is used to represent convection in all four domains..

Two different urban canopy models are used in this study to find the best configuration that will reproduce the extreme rainfall days that occurred in 2014. Urban Canopy parameterizations used in this study are:

- Single layer UCM^37^: is referred to as WRF-SUCM.
- Building Effect Parameterization^38^: is referred to as WRF-MUCM.
- Standard WRF-Noah^58^: is referred to as WRF-NoUCM.

In the original Noah land surface model (LSM), urban physical processes are not parameterized explicitly, but it tries to reproduce resultant urban effects by modifying the prescribed values of vegetation and soil parameters for an urban patch.

WRF-MUCM has a multilayer representation of the urban region where buildings act as a source and sink for heat distribution^38^ whereas WRF-SUCM does not consider variation in building height and building density of model grid cell (details are in the Supplementary Notes). A spin-up time of 6 hours is used before the start of each simulation.

## Electronic supplementary material

Supplementary Information
