# Supplementary material for: Increased Spatial Variability and Intensification of Extreme Monsoon Rainfall due to Urbanization
Source: Sci Rep. 2018 Mar 2;8:3918. doi: 10.1038/s41598-018-22322-9 (PMC5834499; doi:10.1038/s41598-018-22322-9)
Supplement: Supplementary file 1 — Supplementary Information [file 41598_2018_22322_MOESM1_ESM.doc]

**Supplementary Information for:**

**Increased Spatial Variability and Intensification of Extreme Monsoon Rainfall due to Urbanization**

Supantha Paul^1^, Subimal Ghosh^1, 2,*^, Micky Mathew^2^, Anjana Devanand^1^

Subhankar Karmakar^1,3^ and Dev Niyogi^1,4^

^1^Interdisciplinary Program in Climate Studies, Indian Institute of Technology Bombay,

Mumbai – 400076, India

| ^2^Department of Civil Engineering, Indian Institute of Technology Bombay,  Mumbai – 400076, India  ^3^Centre for Environmental Science and Engineering, Indian Institute of Technology Bombay,  Mumbai – 400076, India |
| --- |
|  |

^4^Department of Earth, Atmospheric, and Planetary Sciences and Department of Agronomy- Crops, Soils, Water Sciences, Purdue University, West Lafayette, IN 47906, USA

^*^Department of Civil Engineering, Indian Institute of Technology Bombay, Powai, Mumbai – 400 076, India, Email: [subimal@civil.iitb.ac.in](mailto:subimal@civil.iitb.ac.in); Phone: +91 22 2576 7319; Fax: +91 22 2576 7302

# Supplementary Text

# WRF with Single layer UCM (WRF-SUCM)

In WRF model it is possible to couple an Urban Canopy Model (UCM) with the land surface model to simulate the turbulent heat and momentum fluxes as well as other urban impacts on urban hydro-climatology. The motions around an individual building or buildings can be explicitly resolved with computational fluid dynamics (CFD) models, which are computationally intensive^S1^. To avoid such micro-scale simulations, WRF-UCM coupled models are parametrized to account for the subgrid scale urban variability. However, these parameterization schemes vary considerably in their degrees of freedom to treat urban processes^37^. In this study, we have coupled a single-layer urban canopy model and multilayer Urban Canopy model with the Noah land surface model (LSM). The UCMs consider the 3-D geometry of building and roads to represent the radiation trapping and wind shear in the urban canopy.

The basic function of Noah LSM is to provide surface sensible, latent heat flux, and surface skin temperature as lower boundary conditions for the atmospheric model. In order to supplement the urban effects to this LSM, urban fraction data is supplied that represents the proportion of impervious surfaces. For a given WRF grid cell, the Noah model calculates surface fluxes and temperature for vegetated urban areas (trees, parks, etc.), whereas fluxes for anthropogenic surfaces are provided by the UCM^37^.

Single-layer urban canopy model was (SUCM) developed by Kusaka et al^S2^ and Kusaka and Kimura^S3^. Urban geometry is represented by infinitely long street canyons with a three dimensional nature of urban surfaces. In these canyons, different states of radiation are onsidered and an exponential wind profile is prescribed. Different prognostic variables include surface skin temperatures at the roof, wall, and road (calculated from the surface energy budget) and temperature profiles within roof, wall, and road layers. Diurnal variations of the anthropogenic heating are added to the sensible heat flux from the urban canopy layer^37^.

# WRF with Multi-layer urban canopy (WRF-MUCM)

The multi-layer UCM^38^, allows for a direct interaction of the urban canopy with the planetary boundary layer. BEP recognizes vertical distribution of sources and sinks of heat, moisture, and momentum induced by the buildings through the urban canopy layer. The effects of urban surfaces (vertical and horizontal) on momentum, turbulent kinetic energy (TKE), and potential temperature are taken into account. The Noah–BEP model is available with two turbulence schemes: Bougeault and Lacarrere (1989)^55^ and Mellor–Yamada–Janjic^S4^ in WRF. In the standard version of BEP^38^, the internal temperature of the buildings is generally kept constant. In its more complex form, heat exchanges between interior of the building walls and exterior environment is considered. In this study, we have selected standard version of BEP within WRF framework (WRF-MUCM).

**Role of Urbanization in extreme events**

Urbanization introduces two predominant factors: it introduces additional instability in both horizontal and vertical direction in an urban area in the form of heat, momentum, and turbulent kinetic energy. This additional energy can transport more water vapor in the atmosphere. Secondly, the urban boundary layer (UBL) heats up due to anthropogenic heat and turbulent heat fluxes^S11^ and can carry greater amount of moisture if there is a nearby moisture source.

WRF-NoUCM considers a zero-order effect of urban surfaces^S12^. It uses constant values for roughness length (turbulence), albedo and heat capacity for all types of urban surfaces (walls, roofs, and roads) with a reduced green vegetation fraction over urban area. Hence, none of the factors discussed above are explicitly considered in WRF-NoUCM simulations. A single-layer urban canopy model (WRF-SUCM) considers urban surface as a two-dimensional, symmetrical street canyons with infinite length. Anthropogenic heat (AH) and its diurnal profiles are included and added to the sensible heat flux from the street canyon^S13^. However, it is unable to consider additional instability in both horizontal and vertical direction in an urban area in the form of turbulent kinetic energy due to presence of buildings.

WRF-MUCM considers a direct interaction between the building and the planetary boundary layer (PBL). In this scheme, the UCM considers buildings and their density in terms of height and street canyons in terms of widths. The presence of a buildings generates vortices around them that help to convert the mean kinetic energy to turbulent energy. In general, the BEP(building effect parameterization) in MUCM consists of (i) drag forces induced by buildings, (ii) enhancement of the transformation of mean kinetic energy into turbulent kinetic energy(TKE) and (iii) modification of the heat fluxes due to shadowing and radiation trapping effects(Supplementary Figure 11). BEP considers vertical distribution of sources and sinks of heat, moisture, and momentum through the whole urban canopy layer which substantially impacts the thermodynamic structure of the urban roughness sub-layer and hence the lower part of the urban boundary layer. The details of the differences in simulated processes by WRF-NoUCM, WRF-SUCM and WRF-MUCM are presented in Supplementary Figure 11. The additional turbulent energy due to spatially varying building and road structures within city results into realistic representation of urban micro-climate with increased spatial variability of precipitation.

To resolve the finer scale turbulent processes (e.g. vertical eddies) in an urban area due to building and street structures, it is necessary to simulate the urban precipitation with a very high vertical as well as horizontal resolution. It is also necessary to consider several numerical layers within the urban canopy. In our WRF simulation, additional layers are allocated within the PBL (total 42 vertical layers are considered instead of conventional 30 layers).

**Supplementary Table 1** Correlation of rainfall data as obtained from the rain gauge station at Santacruz, and nearby Automatic Weather Stations (AWS)

| **BMC station** | **AWS Station** | **Distance (km)** | **Correlation** | |
| --- | --- | --- | --- | --- |
|  |  |  | **2014** | **2015** |
| Santacruz | Bandra | 3.57 | 0.81 | 0.72 |
|  | BKC | 3.76 |  |  |
|  | Dharavi | 4.80 |  |  |
|  | Andheri | 6.32 |  |  |
|  | BMC Ward Office F North | 6.10 |  |  |

**Supplementary Table 2** Comparison of rainfall (station average, in mm/d) as simulated by WRF with observed data

| **Extreme Event**  **Date** | **Observed Rainfall(mm)** | **Simulated Rainfall (WRF-NoUCM)** | **Simulated Rainfall(WRF-SUCM)** | **Simulated Rainfall**  **(WRF-MUCM)** |
| --- | --- | --- | --- | --- |
| July 11^th^ | 96.30 | 64.48 | 68.94 | 102.90 |
| July 15^th^ | 126.90 | 78.08 | 109.98 | 104.00 |
| July 31^st^ | 113.60 | 76.00 | 79.85 | 89.80 |
| August 31^st^ | 96.80 | 58.00 | 59.78 | 81.00 |

**Supplementary Table 3** Physical parameterization schemes used for sensitivity analysis

| **SL No.** | **Physical Sensitivity scheme** |
| --- | --- |
| Cumulus  Schemes | Kain-Fritsch (new Eta) scheme^51^ |
|  | Betts-Miller-Janjic scheme^S4^ |
|  | Grell-Freitas ensemble scheme ^S5^ |
|  | Grell 3D ensemble scheme ^S6, S7^ |
|  | Modifed Tiedtke scheme (ARW only)^S8, S9^ |
| PBL schemes | Bougeault and Lacarrere (BouLac) PBL^55^ |
|  | Mellor-Yamada-Janjic TKE scheme(MYJ scheme)^S10^ |

**Supplementary Reference**

- 1. Chen, F., Kusaka, H., Tewari, M., Bao, J.W. & Harakuchi, H. Utilizing the coupled WRF/LSM/urban modeling system with detailed urban classification to simulate the urban heat island phenomena over the Greater Houston area.  *Proc. Fifth Conference on Urban Environment, Vancouver, BC, Canada, Amer. Meteor. Soc*., *paper* **9.11**(2004).
  2. Kusaka, H., Kondo, H., Kikegawa, Y. & Kimura, F. A. simple single layer urban canopy model for atmospheric models: comparison with multi-layer and slab models. *Bound-Lay Meteorol* **101**, 329–358(2001).
  3. Kusaka, H. & Kimura, F. Coupling a single-layer urban canopy model with a simple atmospheric model: impact on urban heat island simulation for an idealized case. *J Meteor Soc Japan* **82**, 67–80(2004).
  4. Janjic, & Zavisa, I. The step-mountain eta coordinate: further development of the convection, viscous sublayer, and turbulent closure schemes. *Mon Wea Rev* **122,** 927–945(1994).
  5. Grell, G. A. & Freitas, S. R. A scale and aerosol aware stochastic convective parameterization for weather and air quality modeling, *Atmos. Chem. Phys.*, **14**, 5233-5250, doi:10.5194/acp-14-5233-2014(2014).
  6. [Grell, G. A. Prognostic Evaluation of Assumptions Used by Cumulus Parameterizations. *Mon. Wea. Rev.*, **121**, 764–787(1993).](http://www2.mmm.ucar.edu/wrf/users/phys_refs/CU_PHYS/Grell3D_part1.pdf)
  7. [Grell, G. A, D. Devenyi. A generalized approach to parameterizing convection combining ensemble and data assimilation techniques. *Geophys. Res. Lett.*, **29**, 1693](http://www2.mmm.ucar.edu/wrf/users/phys_refs/CU_PHYS/Grell3D_part2.pdf)(2002).
  8. [Tiedtke, M. A comprehensive mass flux scheme for cumulus parameterization in large–scale models. *Mon. Wea. Rev.*, **117**, 1779–1800(1989).](http://www2.mmm.ucar.edu/wrf/users/phys_refs/CU_PHYS/Tiedtke_part1.pdf)
  9. [Zhang, Chunxi, Yuqing Wang, and Kevin Hamilton. Improved representation of boundary layer clouds over the southeast pacific in ARW–WRF using a modified Tiedtke cumulus parameterization scheme. *Mon. Wea. Rev.*, **139**, 3489–3513(2011).](http://www2.mmm.ucar.edu/wrf/users/phys_refs/CU_PHYS/Tiedtke_part2.pdf)
  10. [Janjic, Zavisa I. The Step–Mountain Eta Coordinate Model: Further developments of the convection, viscous sublayer, and turbulence closure schemes. *Mon. Wea. Rev.*, **122**, 927–945(1994).](http://www2.mmm.ucar.edu/wrf/users/phys_refs/PBL/MYJ.pdf)
  11. Oke, T.R. The urban energy balance. *Prog in Phys Geog.*, **12,** 471–508(1988).
  12. Liu, Y. B., et al. Verification of a mesoscale data-assimilation and forecasting system for the Okalahoma city area during the joint urban 2003 field project. *J. Appl. Meteorol. Climatol*., **45,** 912–929(2006).
  13. Chen, F., et al. The integrated WRF/urban modelling system: Development, evaluation, and applications to urban environmental problems. *Int J Climatol*, **31**, 273–288(2011a).

# Supplementary Figures

**
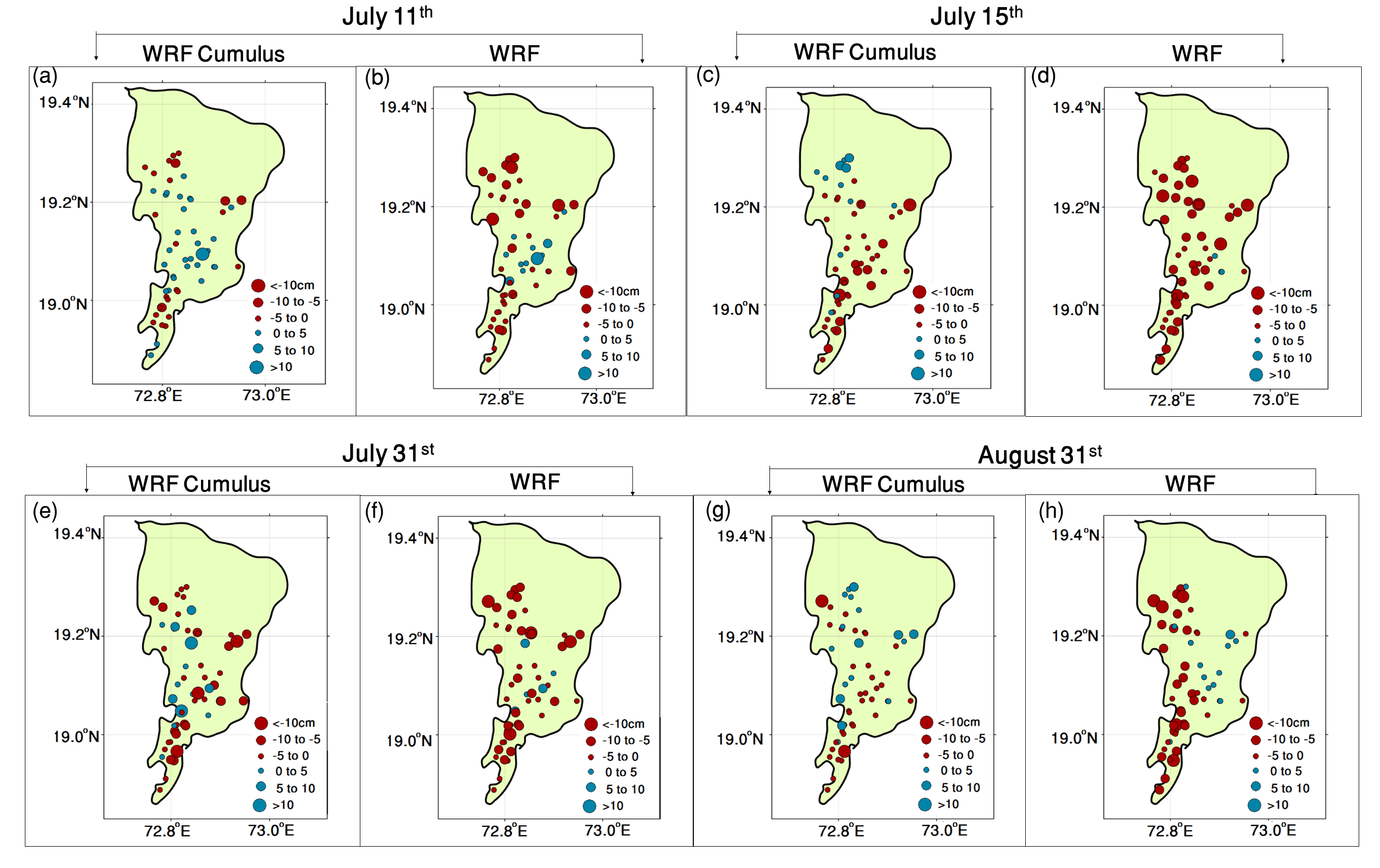
**

**Supplementary Figure 1** Difference between observed cumulative rainfall and WRF simulation for the extreme days in 2014. WRF-MUCM simulation with cumulus schemes(a, c, e , g) and WRF-MUCM simulation without convective parameterizations (b, d , f , h) for July 11th , July 15th, July 31st and August 31st are presented. Maps are prepared with ArcGIS 10.1(http://www.esri.com/news/arcnews/spring12articles/introducing-arcgis-101.html). The shape files of maps are derived from Mumbai Metropolitan Region Development Authority (MMRDA).


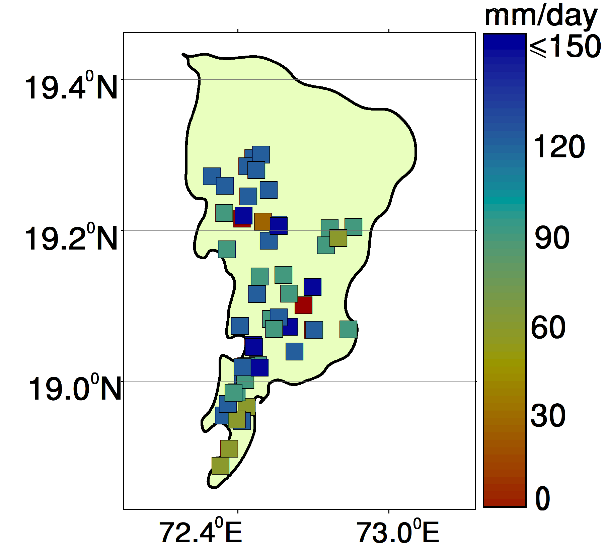

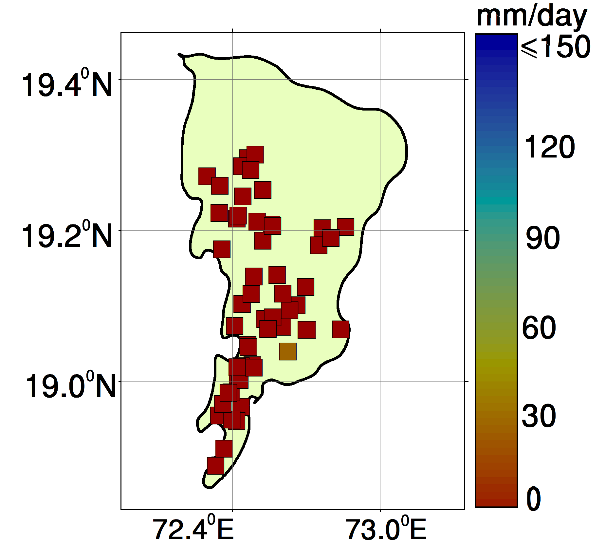

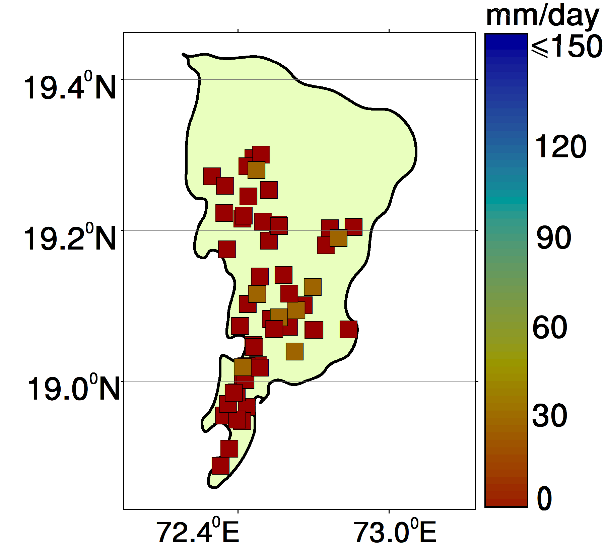

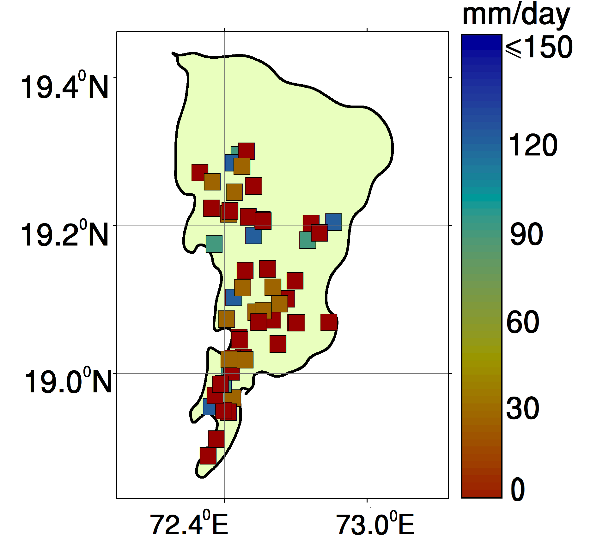

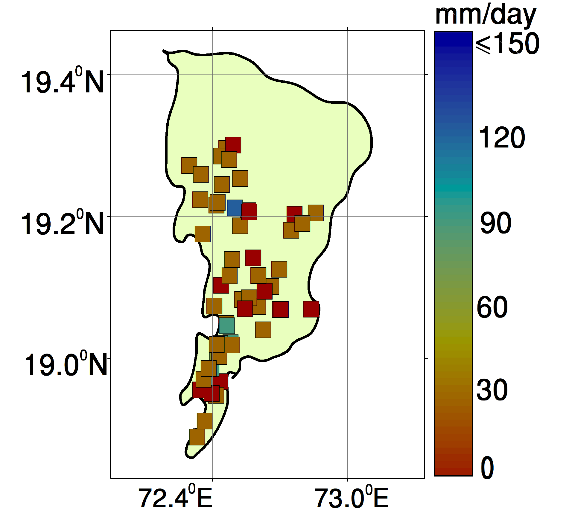

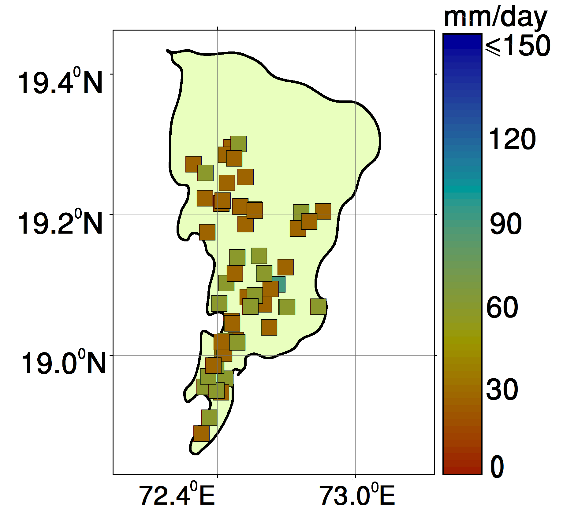

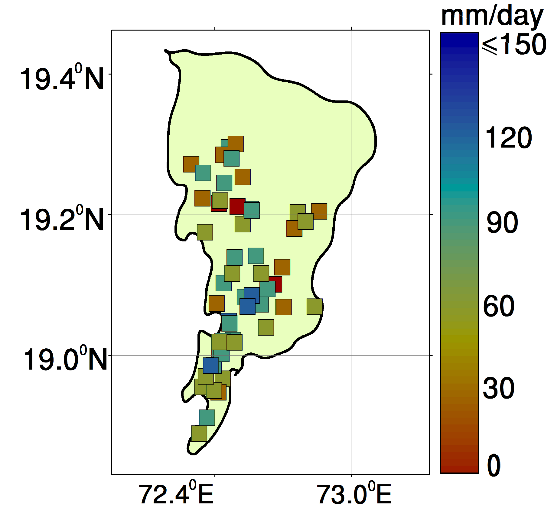

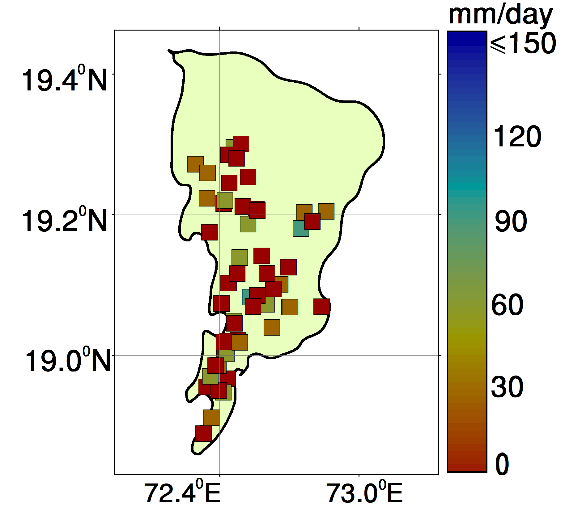

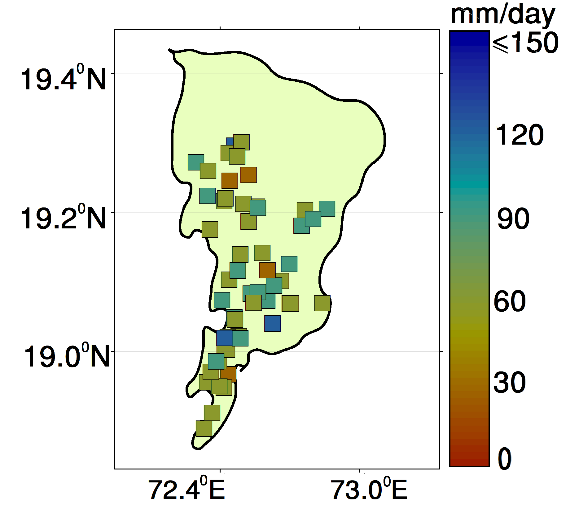

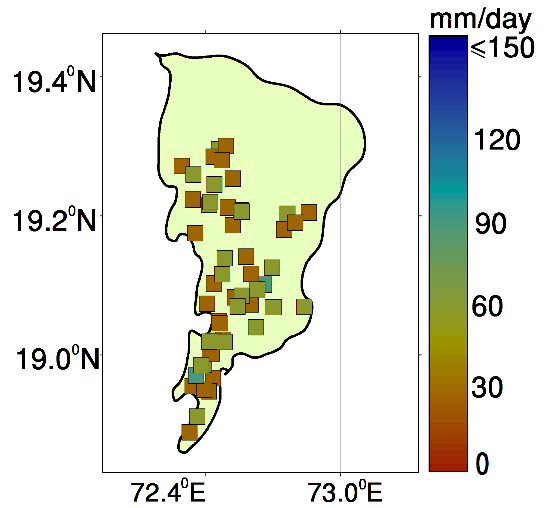

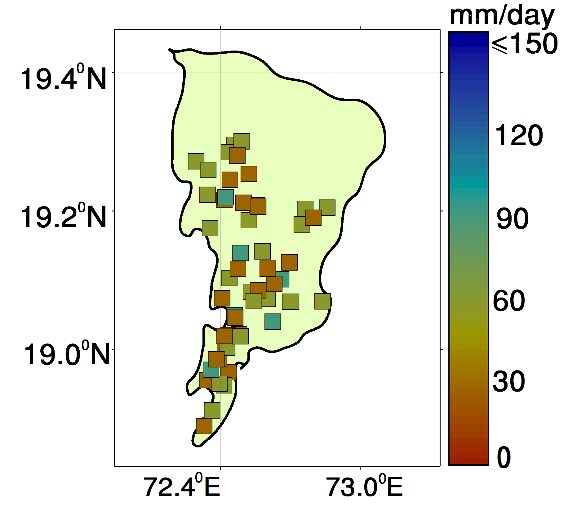

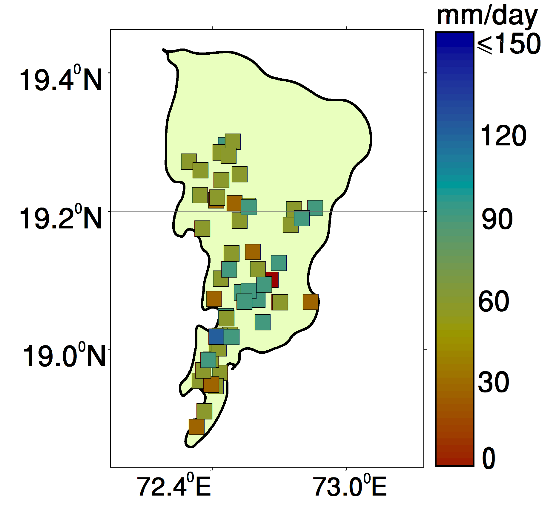

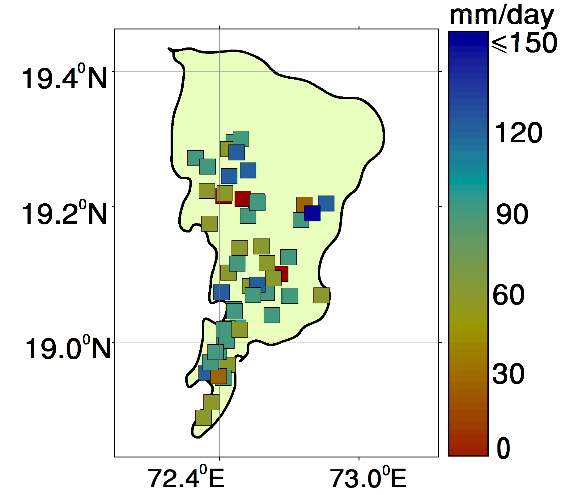

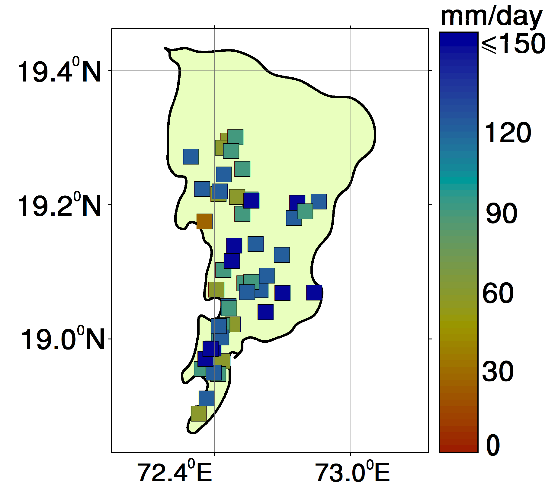

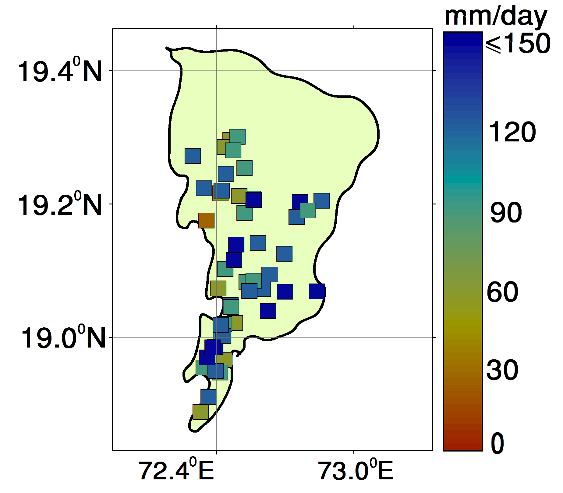

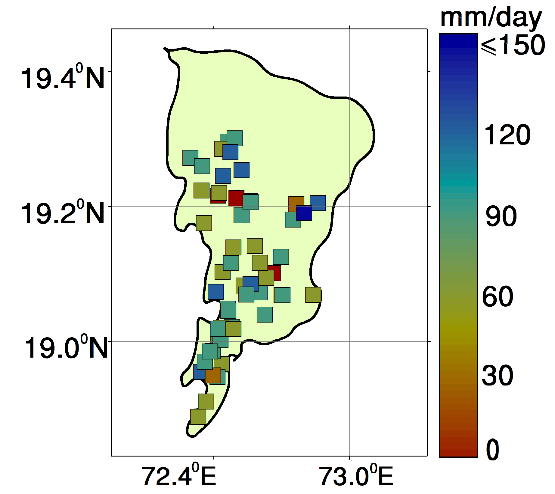

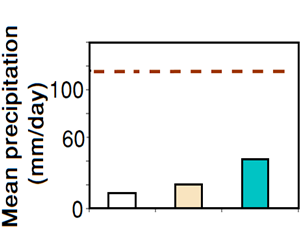

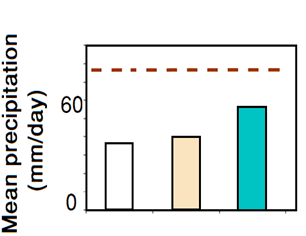

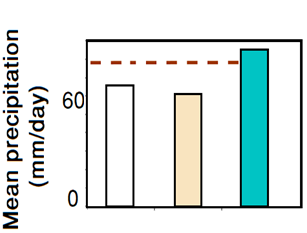

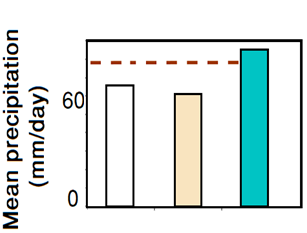


(a)

(b)

(c)

(d)

(e)

(f)

(g)

(h)

(i)

(j)

(k)

(l)

(m)

(n)

(o)

(p)

(q)

(r)

(s)

(t)

**Supplementary Figure 2** Simulated rainfall for extreme days for the year 2015for June 18^th^,


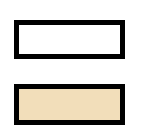

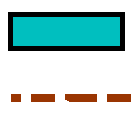


WRF NOUCM

WRF SUCM

WRF MUCM

Observed

June 20^th^, June 21^st^ and June 23^rd^.

Observed precipitation over 55-AWS stations (Fig (a), (f), (k) and (p)) and simulated precipitation over the same stations with WRF-NoUCM(Fig (b), (g), (l) and (q)); WRF-SUCM(Fig (c), (h), (m), and (r)); and WRF-MUCM(Fig (d), (i), (n) and(s)) are presented in first four columns. Spatially averaged precipitation are presented in Fig (e), (j), (o) and (t). Fig(a)-Fig(d), Fig(f)-Fig(i), Fig(k)-Fig(n) and Fig(p)-Fig(s) are prepared with ArcGIS10.1 (http://www.esri.com/news/arcnews/spring12articles/introducing-arcgis-101.html). Fig (e), (j), (o) and (t) are prepared with Matlab R2015b https://in.mathworks.com/products/new_products/release2015b.html). The shape files of maps are derived from Mumbai Metropolitan Region Development Authority (MMRDA).


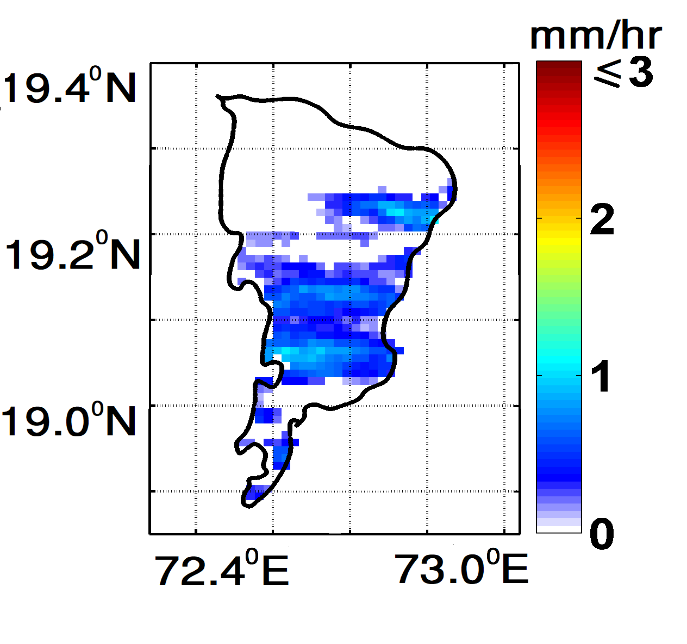

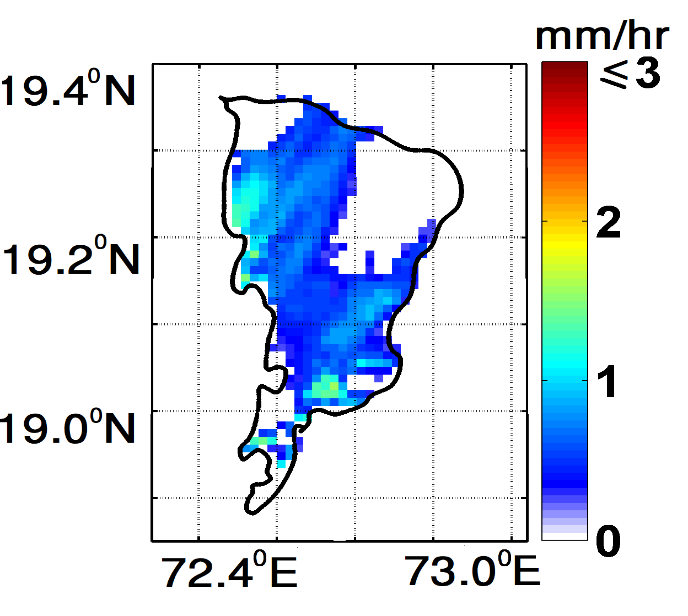


(a)

(b)

**Supplementary Figure 3** Statistically significant differences of hourly rainfall on extreme days computed between the simulations obtained from WRF-MUCM and WRF-No-UCM. They are presented for 2014 (a) and 2015 (b). Figures are prepared with Matlab R2015b https://in.mathworks.com/products/new_products/release2015b.html). The shape files of maps are derived from Mumbai Metropolitan Region Development Authority (MMRDA).


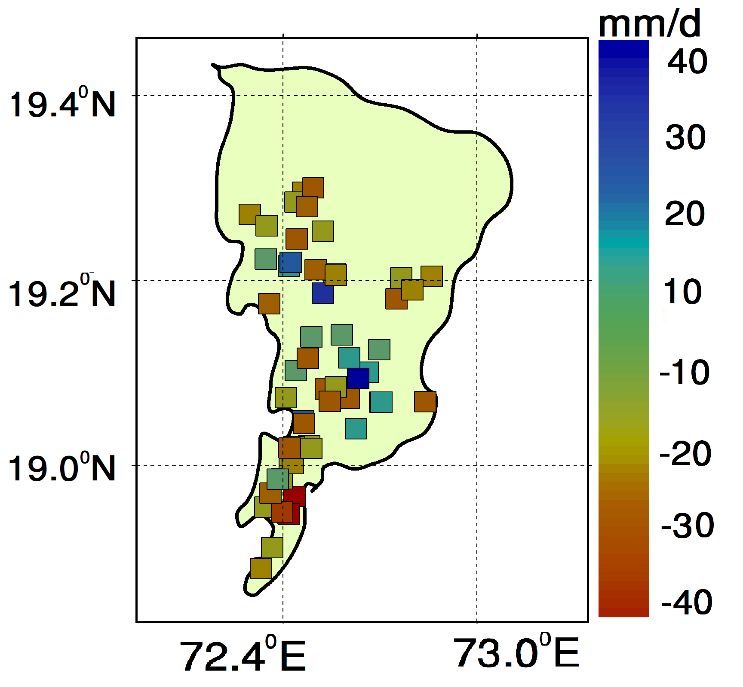

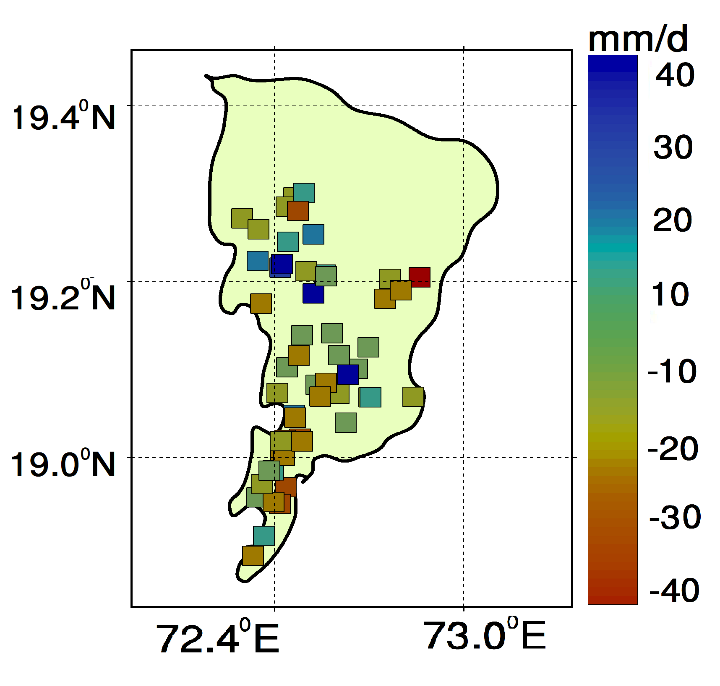

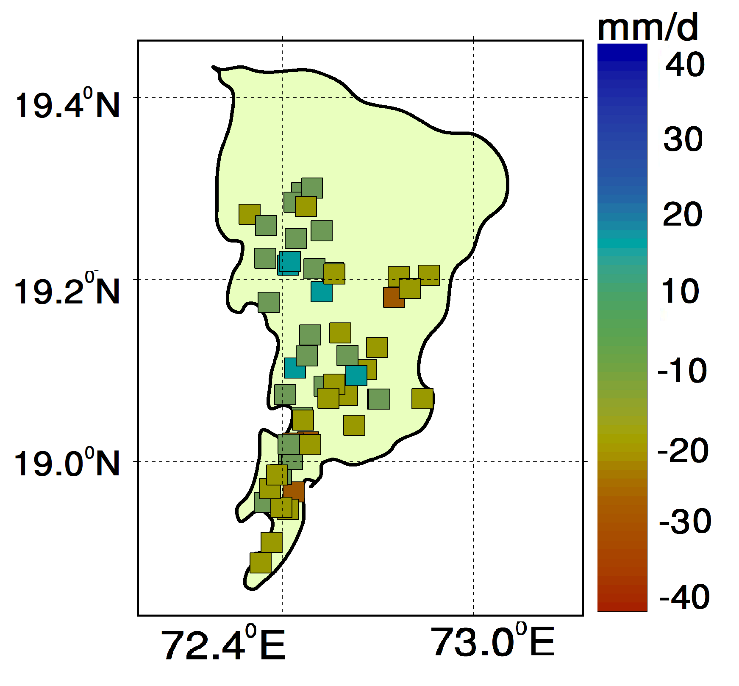

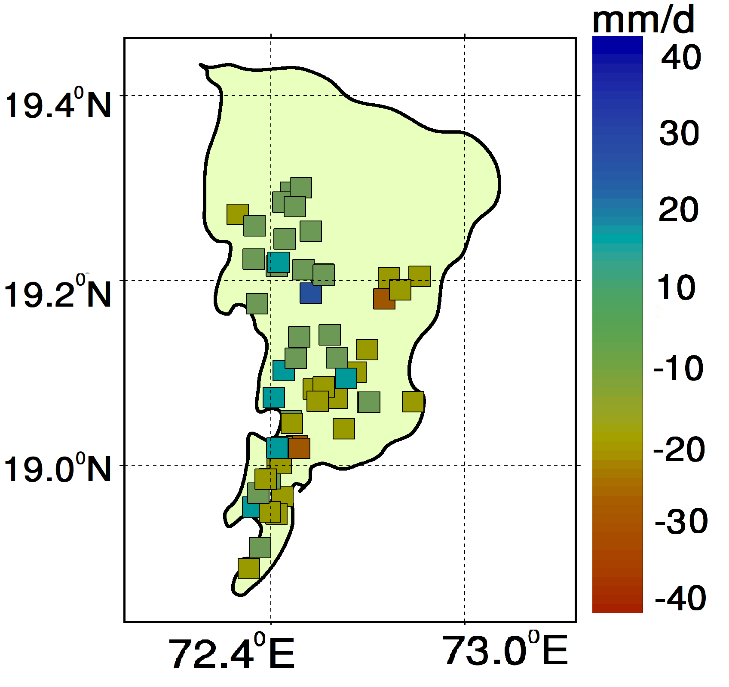

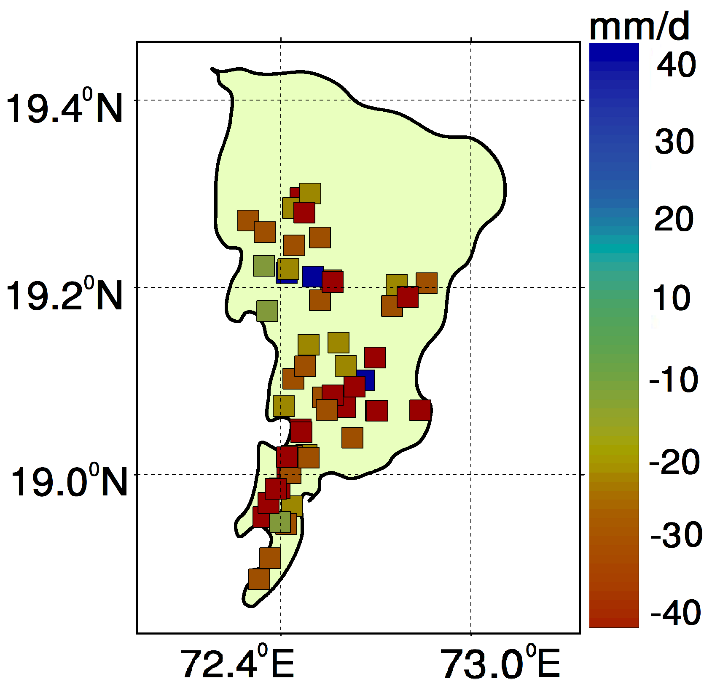

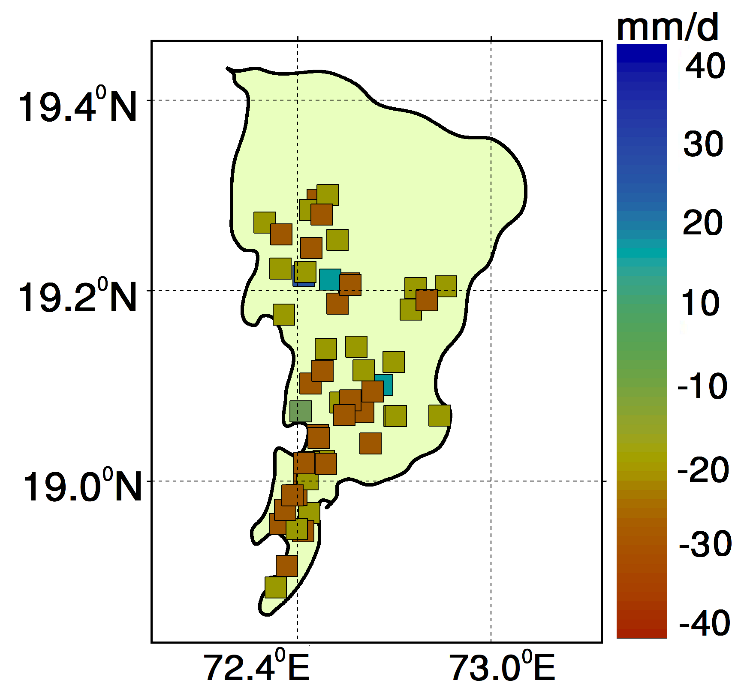

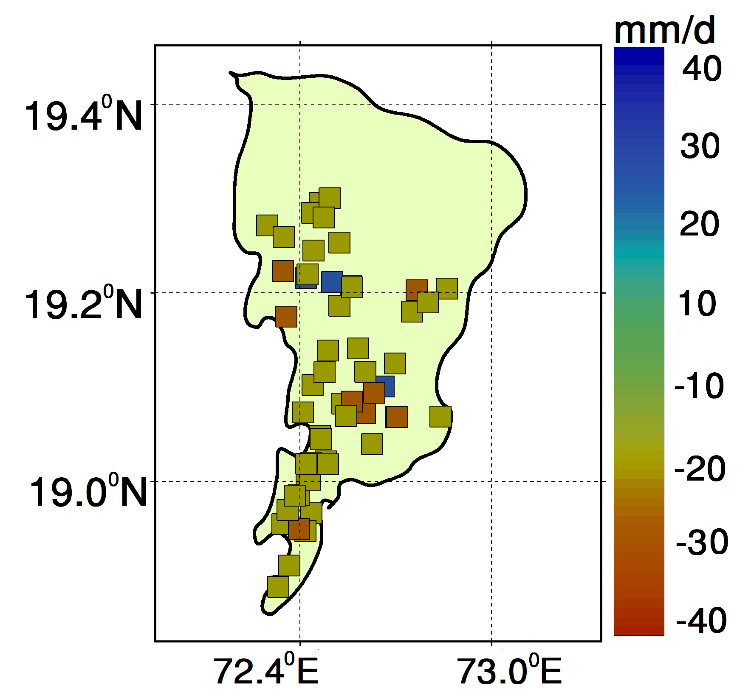

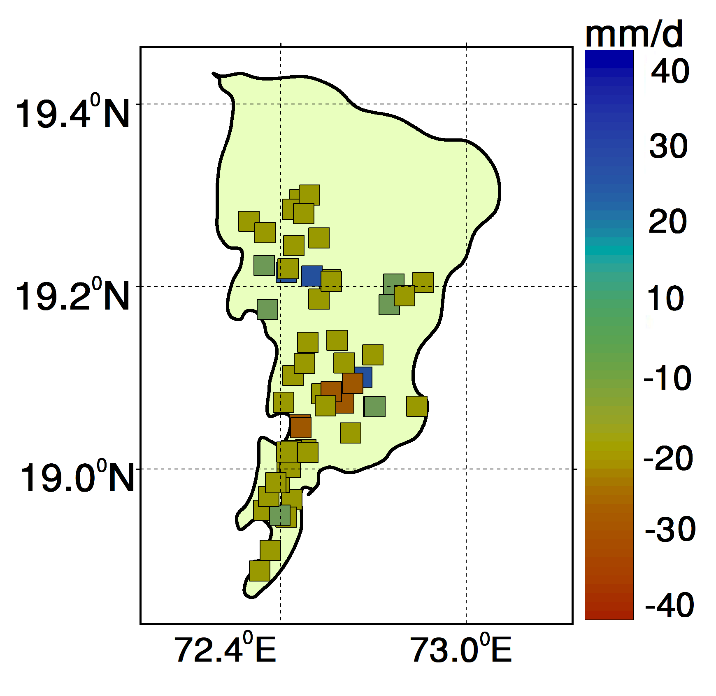


(a)

(b)

(c)

(d)

(e)

(f)

(g)

(h)

**Supplementary Figure 4** Comparison of errors in simulated precipitation as obtained from WRF-MUCM for multiple spatial resolution. Figs (a), (b), (c) and (d) present the mean daily error for 2014 extremes days for the simulations at the spatial resolutions of 27km, 9 km, 3 km and 1 km, respectively . The same for 2015 are presented in Figs (e)-(h) in the same order. Figures are prepared with Matlab R2015b https://in.mathworks.com/products/new_products/release2015b.html). The shape files of maps are derived from Mumbai Metropolitan Region Development Authority (MMRDA).


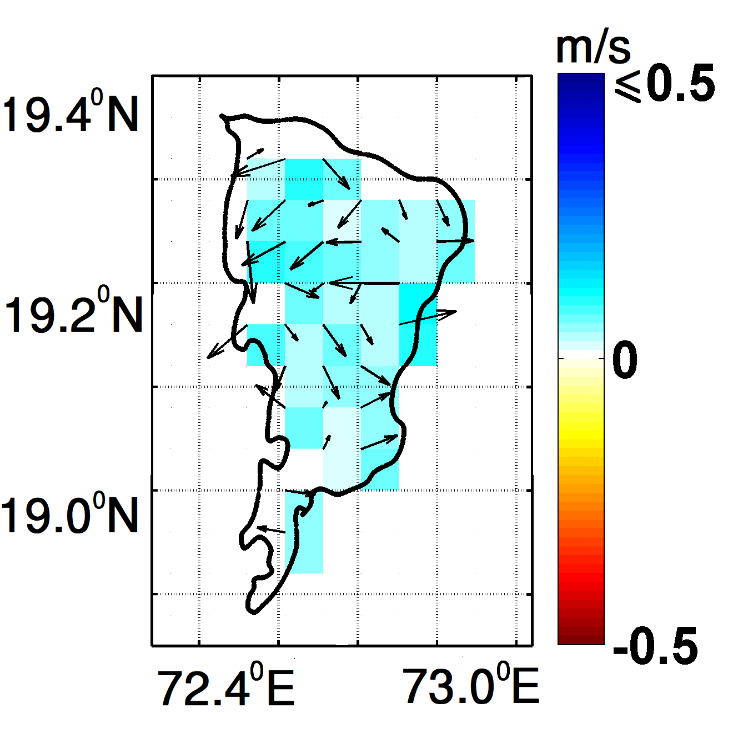

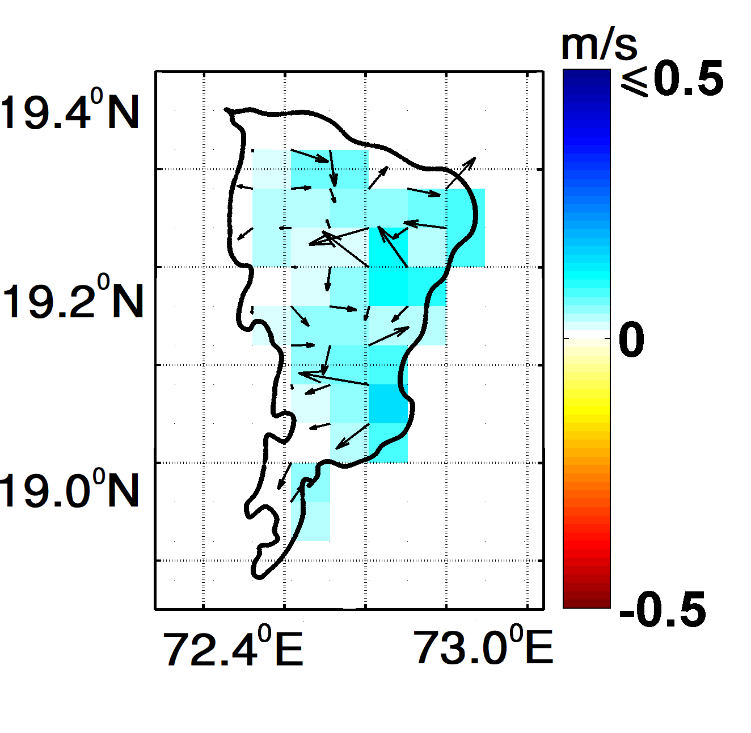

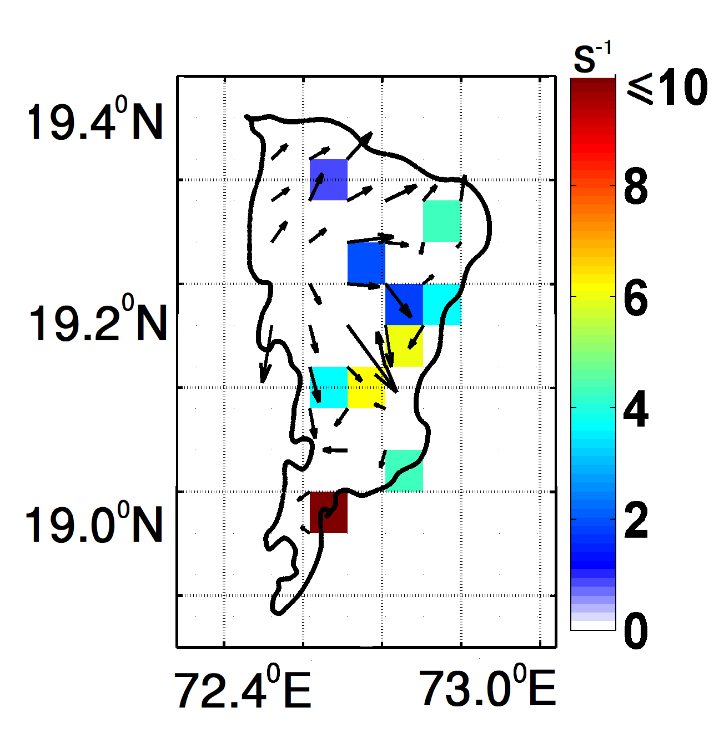

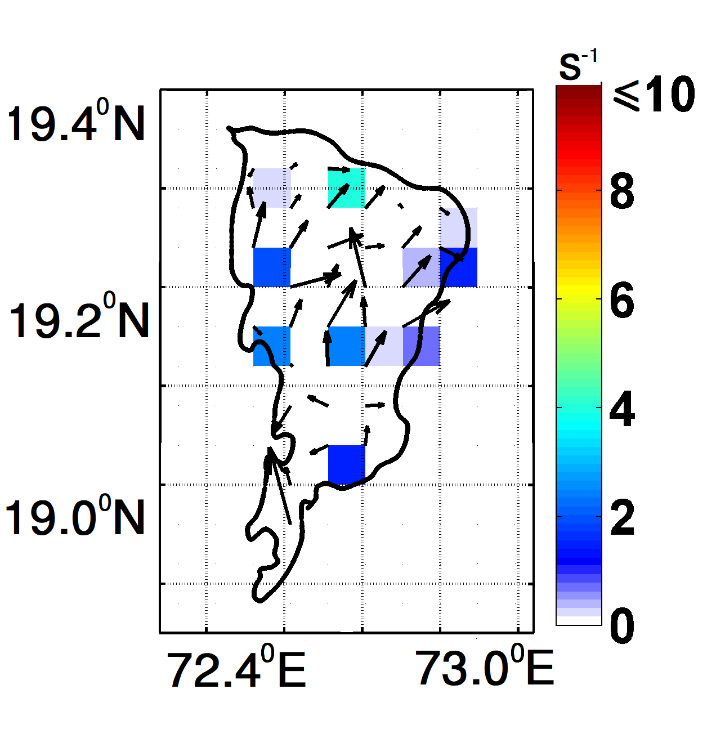

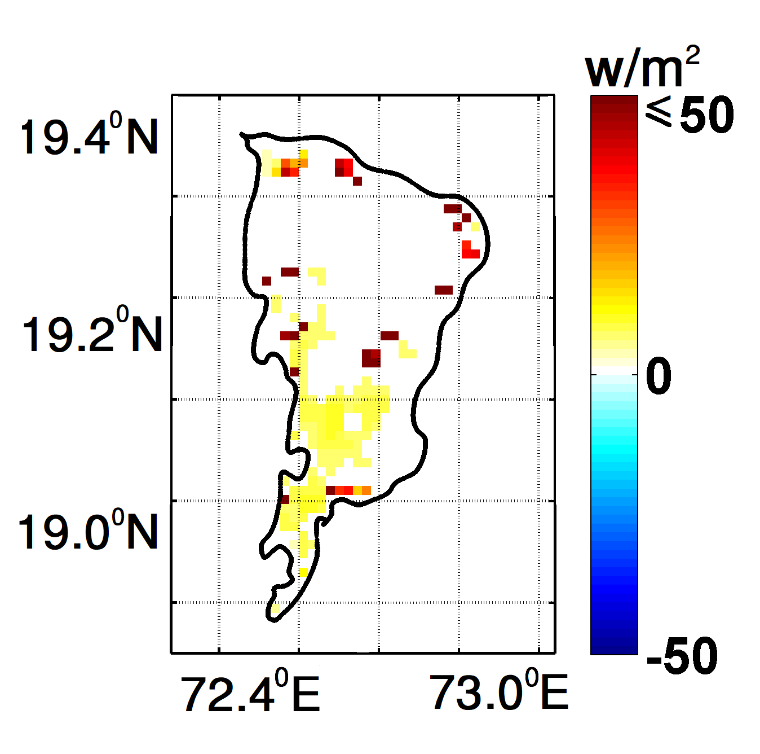

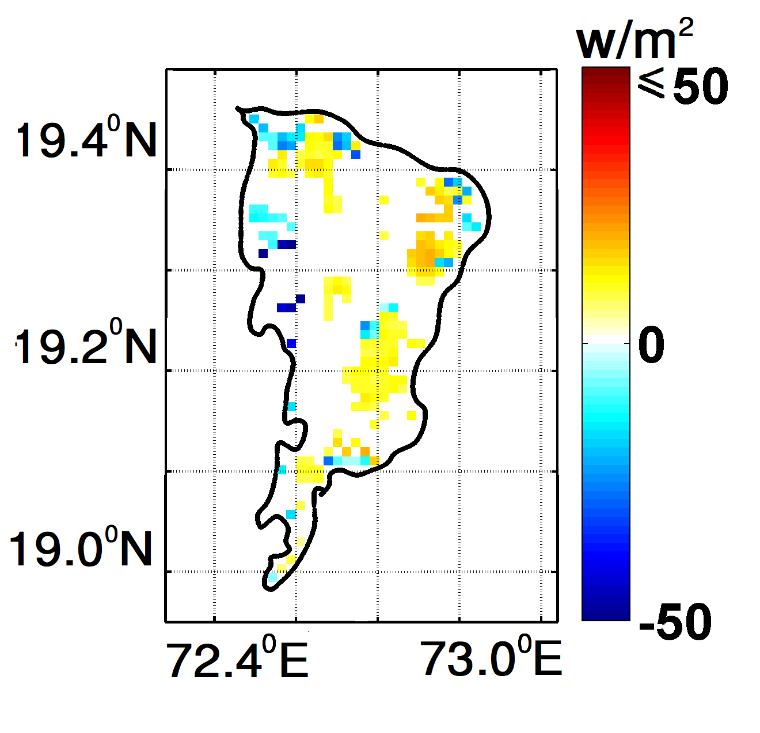


(a)

(b)

(c)

(d)

(e)

(f)

**Supplementary Figure 5** Differences between the simulated meteorological variables as obtained from WRF –MUCM and WRF-NoUCM for extreme days of year 2014 (top row) and 2015 (bottom row). First, second and third column represent wind at 850hPa, moisture convergence and surface heat flux. Figures are prepared with Matlab R2015b https://in.mathworks.com/products/new_products/release2015b.html). The shape files of maps are derived from Mumbai Metropolitan Region Development Authority (MMRDA).


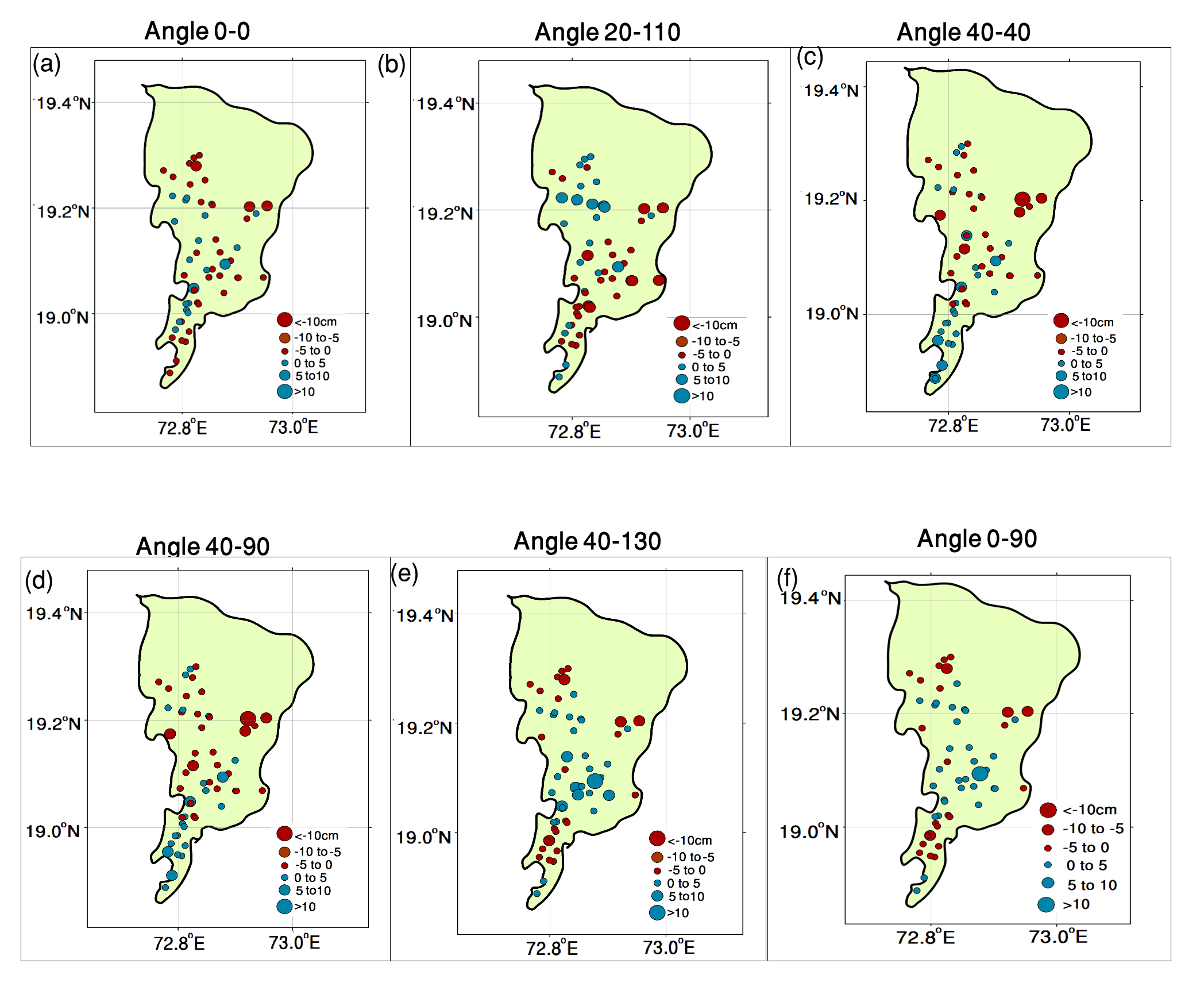


**Supplementary Figure 6** Impact of street alignment on errors in simulated precipitation by WRF MUCM (July 11th, 2014). Maps are prepared with ArcGIS 10.1 (http://www.esri.com/news/arcnews/spring12articles/introducing-arcgis-101.html). The shape files of maps are derived from Mumbai Metropolitan Region Development Authority (MMRDA).


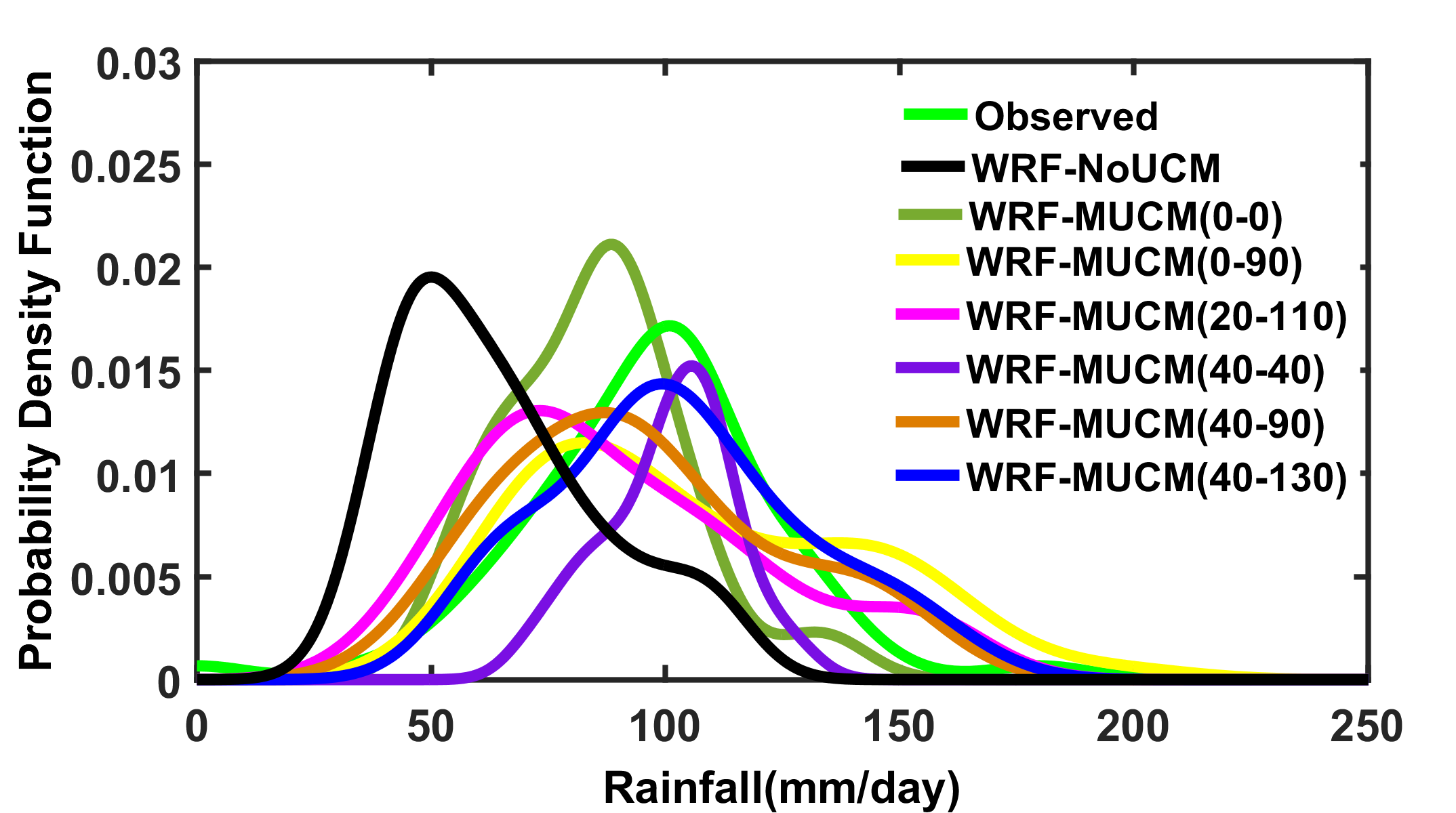


**Supplementary Figure 7** Effect of street alignment on spatial variability of rainfall during extreme days. Numbers in the bracket represent road alignment (in degrees). Figure is prepared with Matlab R2015b (https://in.mathworks.com/products/new_products/release2015b.html).

**
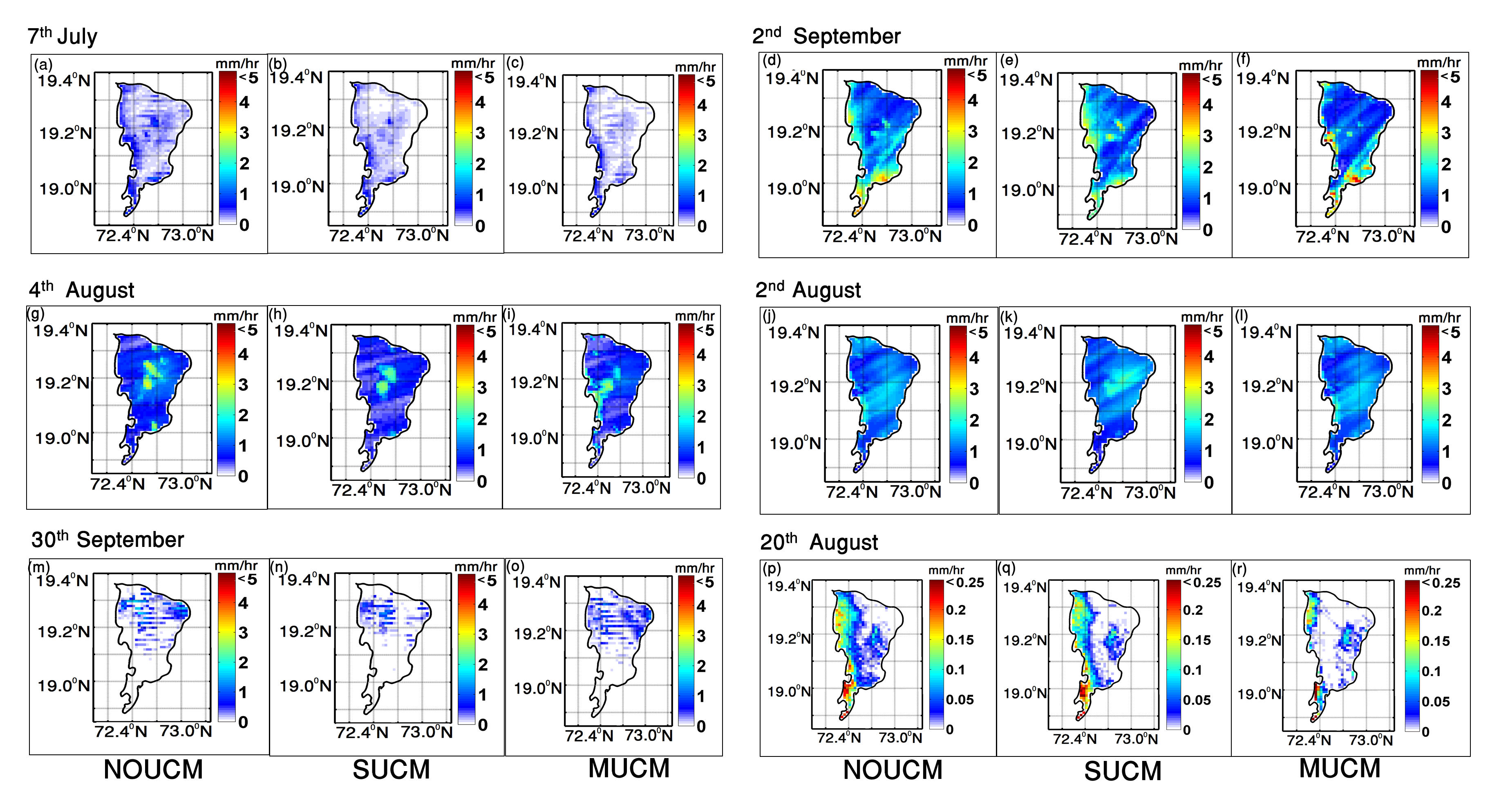
**

**Supplementary Figure 8** Plot of low rainfall days with WRF NoUCM, SUCM and MUCM schemes. Rainfall days with moderately low(40-50mm/day,(a)-(i)), low(20-30mm/day,((j)-(o)) and extremely low(<5mm/day, (p),(q) and(r)) intensity days are plotted. Plots represent hourly mean rainfall days averaged over Mumbai region (mm/hr). Figure is prepared with Matlab R2015b (https://in.mathworks.com/products/new_products/release2015b.html).

**
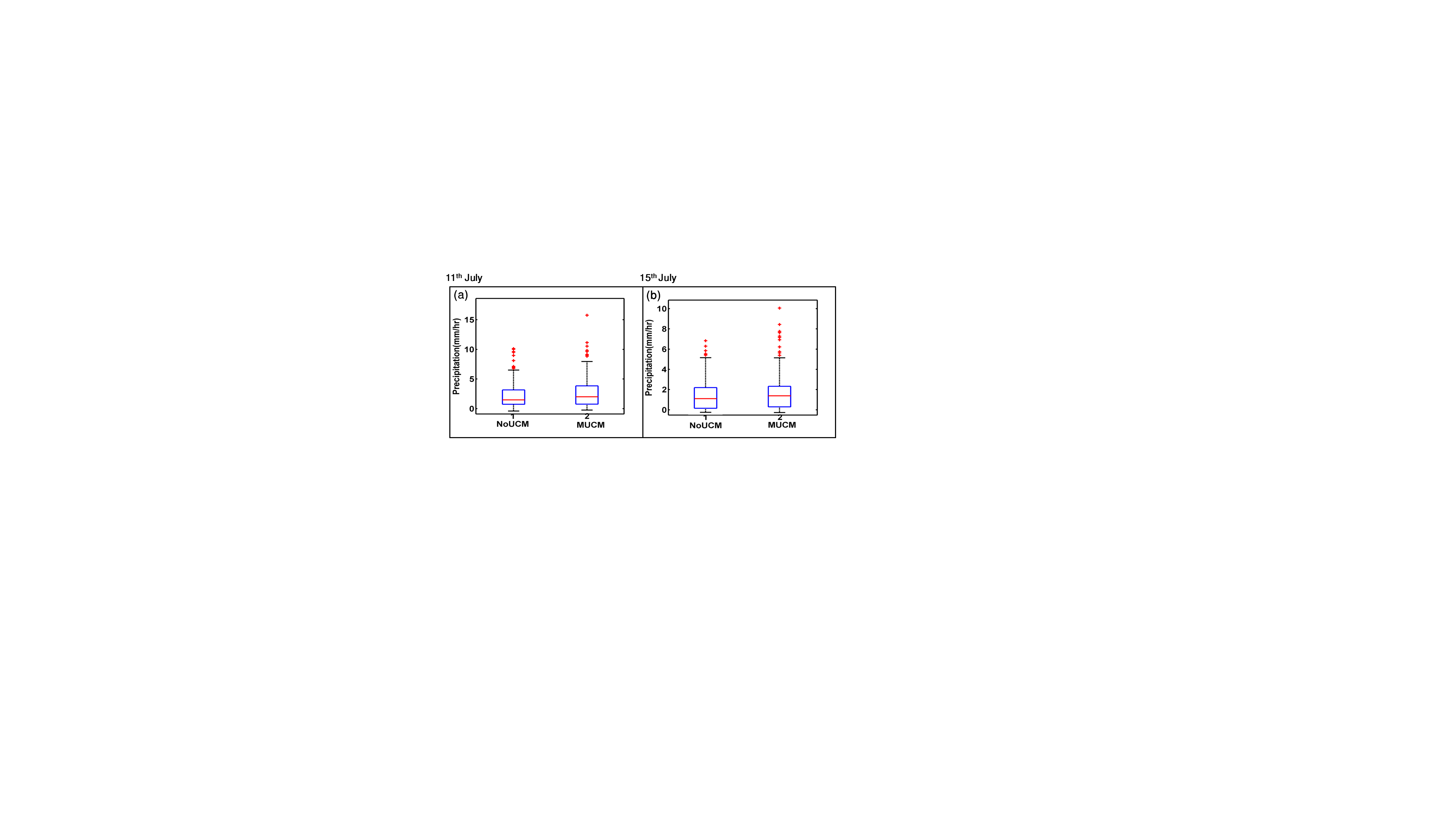
**

**Supplementary Figure 9** Plot of extreme rainfall days with multiple physical parameterization schemes. Additional five(5) Cumulus schemes in combination with two(2) different PBL schemes are used for simulating July 11th and July 15th extreme rainfall days. Box plots are prepared with a composite time series across all parameterization schemes for both MUCM and NoUCM case(Fig.(a)-(b)) . All precipitation data are computed considering all the grid points over Mumbai). Figure is prepared with Matlab R2015b (https://in.mathworks.com/products/new_products/release2015b.html).

**
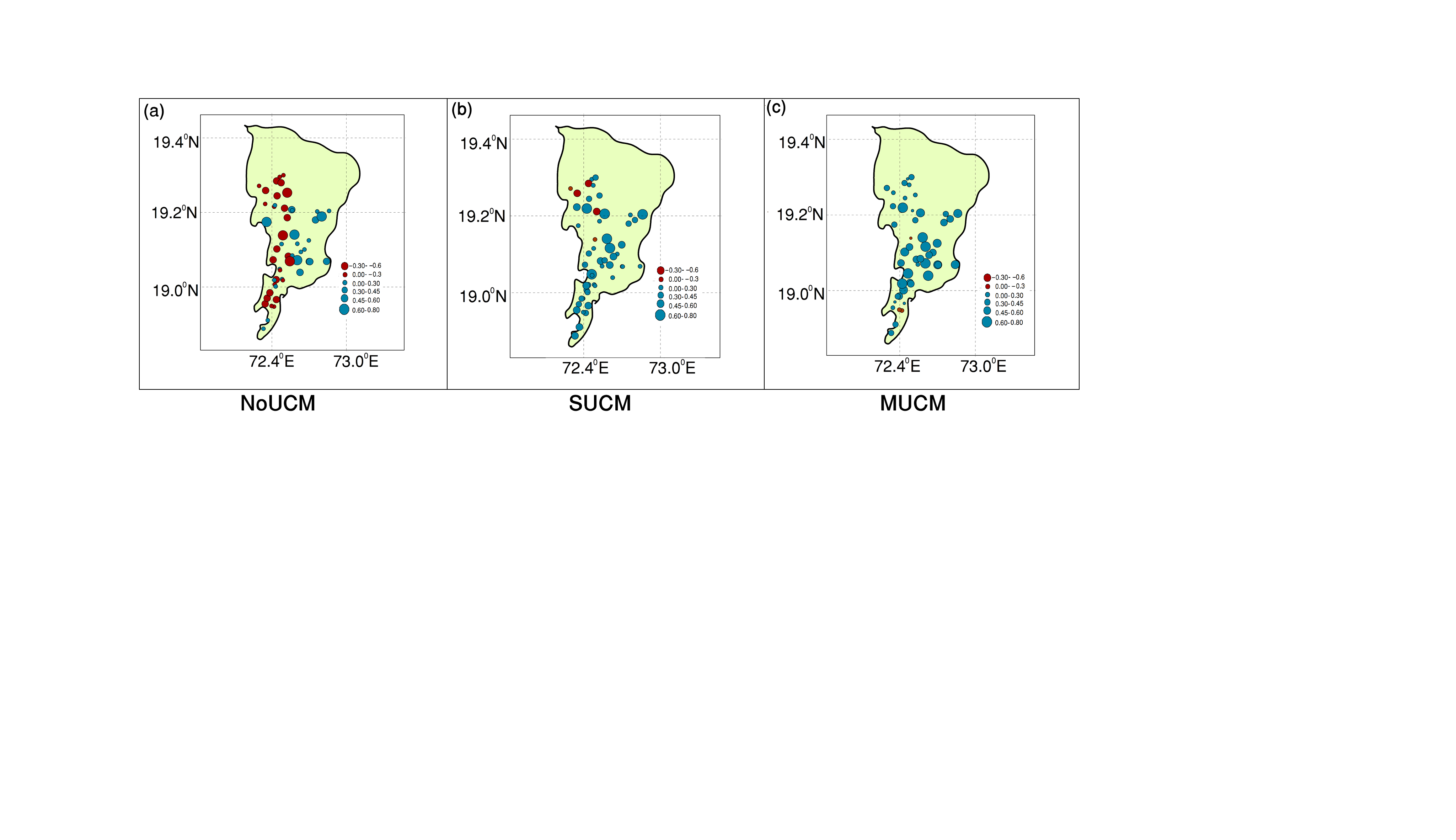
**

**Supplementary Figure 10** Plot of spatial correlation of extreme rainfall days for MUCM, SUCM and NoUCM case(Computed over all the AWS stations over Mumbai). Plots are based on cumulative 6-hr rainfall (mm). Maps are prepared with ArcGIS 10.1 (http://www.esri.com/news/arcnews/spring12articles/introducing-arcgis-101.html). The shape files of maps are derived from Mumbai Metropolitan Region Development Authority (MMRDA).


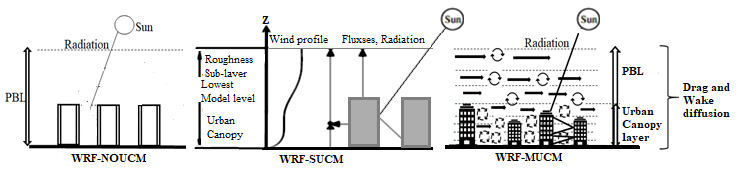


**Supplementary Figure 11**
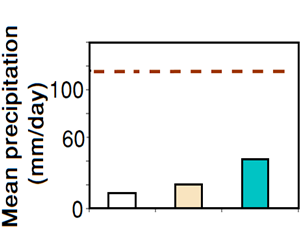

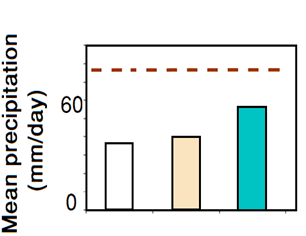

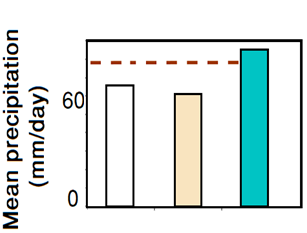

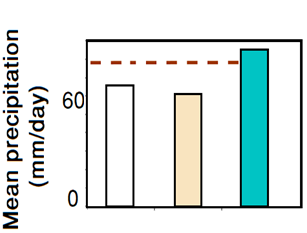
Comparison of WRF-MUCM and WRF-NoUCM
